# Cytokinesis requires localized β-actin filament production by an actin isoform specific nucleator

**DOI:** 10.1038/s41467-017-01231-x

**Published:** 2017-11-16

**Authors:** A. Chen, P. D. Arora, C. A. McCulloch, A. Wilde

**Affiliations:** 1 Department of Biochemistry, University of Toronto, 661 University Ave, Mars Tower West, Toronto, ON Canada M5G 1M1; 2 Matrix Dynamics Group, Faculty of Dentistry, University of Toronto, Room 243, Fitzgerald Building, 150 College Street, Toronto, ON Canada M5S 3E2; 3 Department of Molecular Genetics, University of Toronto, 661 University Ave, Mars Tower West, Toronto, ON Canada M5G 1M1

## Abstract

Cytokinesis is initiated by the localized assembly of the contractile ring, a dynamic actomyosin structure that generates a membrane furrow between the segregating chromosomal masses to divide a cell into two. Here we show that the stabilization and organization of the cytokinetic furrow is specifically dependent on localized β-actin filament assembly at the site of cytokinesis. β-actin filaments are assembled directly at the furrow by an anillin-dependent pathway that enhances RhoA-dependent activation of the formin DIAPH3, an actin nucleator. DIAPH3 specifically generates homopolymeric filaments of β-actin in vitro. By employing enhancers and activators, cells can achieve acute spatio-temporal control over isoform-specific actin arrays that are required for distinct cellular functions.

## Abstract (web summary)

Cytokinesis is initiated by the localized assembly of the contractile ring. Here the authors show that the stabilization and organization of the cytokinetic furrow requires localized β-actin filament assembly at the site of cytokinesis by an actin isoform specific nucleator.

## Introduction

The final stage of cell division, cytokinesis serves to divide one cell into two new daughters, each with a complete genome^1^. An early stage of cytokinesis involves the invagination of the plasma membrane between the segregating chromosomal masses to create the cytokinetic furrow. As cytokinesis progresses, the furrow continues to ingress and to be remodeled until a final membrane fusion event, abscission, generates two independent cells.

A specific actomyosin array, the contractile ring, generates the force required to deform the plasma membrane and drive cytokinetic furrow ingression^2^. However, the detailed principles underlying the temporal and spatial organization of the contractile ring are unclear. For instance, different sources of actin are required for cytokinesis^3^; one source comes from the poles and moves toward the furrow in a process called cortical flow^4–7^, while a second source of actin is produced de novo at the site of furrowing^8^. How each of these mechanisms is regulated and contributes to cytokinesis is unclear.

**Fig. 1:**
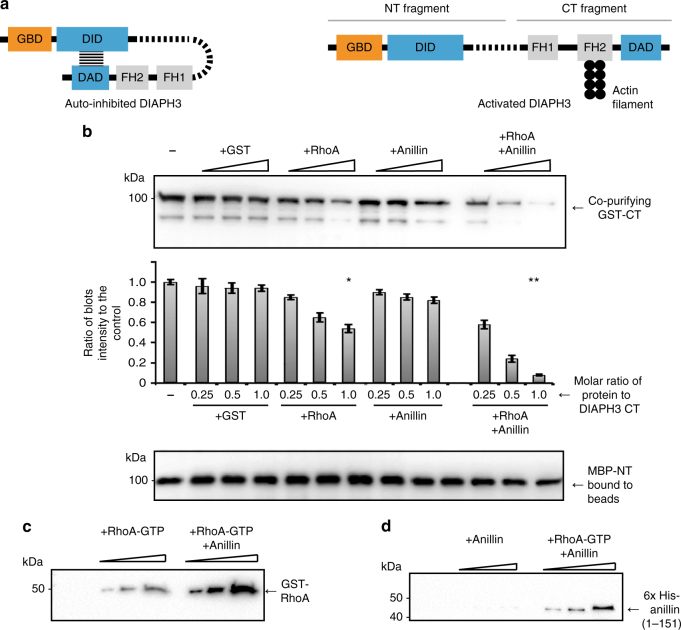
Anillin enhances RhoA-mediated release of DIAPH3 intramolecular auto-inhibition interaction. **a** Schematic of DIAPH3 domain organization in the auto-inhibited and activated states (not to scale). **b** Immunoblots and quantification to determine the amount of GST-DIAPH3-CT and MBP-DIAPH3-NT bound to amylose beads in the presence of increasing concentrations of GST, GST-RhoA, and 6× His-anillin_(1–151 aa)._ Values normalized to the amount of MBP-DIAPH3-NT immobilized on beads. Each binding assay was repeated at least three times. Error bars indicate ± s.e.m. *p = 0.014, **p = 0.0009 compared to GST control, Student’s *t* test. *n* = 3. **c** MBP-DIAPH3-NT and GST-DIAPH3-CT were pre-mixed, then incubated with either GST-RhoA, 6× His-anillin_1–151_, or both. MBP-DIAPH3-NT was re-isolated on amylose beads and immunoblots performed to detect co-purifying RhoA and **d** anillin

The actin at the cytokinetic furrow becomes organized into the actomyosin-rich contractile ring. The assembly of the ring requires exquisite spatio-temporal control including signaling through small GTPases^9–11^, enrichment of specific lipids at the plasma membrane^12–14^ and recruitment of the actomyosin, microtubule, and septin cytoskeletons^1^. The essential cytokinesis factor, anillin, has the potential to integrate many of these elements through direct interaction^15,16^. Anillin is a conserved scaffolding protein that localizes to the cytokinetic furrow, where it interconnects the actin, myosin, septin, and microtubule cytoskeletons with the plasma membrane^16^. In addition, anillin recruits the formin DIAPH3, an actin nucleator, to the cleavage furrow^17^. Some formins exist in an auto-inhibited state resulting from an intramolecular interaction between the diaphanous inhibitory and autoregulatory domains (DID and DAD, respectively)^18^ (Fig. 1a). Activation of diaphanous-related formins involves the binding of an activated G-protein such as RhoA^19,20^. However, G-protein binding alone only partially activates formins, indicating that additional factors are likely required^21,22^.

Here we demonstrate that anillin enhances RhoA-dependent DIAPH3 actin nucleation activity by enhancing the ability of RhoA to relieve the DID–DAD interaction of DIAPH3. Furthermore, we demonstrate that DIAPH3 nucleates only one of the ubiquitously expressed actin isoforms, β-actin but not γ-actin, and that in vitro, DIAPH3 nucleates β-actin homopolymers. During cytokinesis the anillin–DIAPH3 interaction specifically promotes the production of β-actin filaments at the cytokinetic furrow, which underlies stable furrow ingression. In contrast, γ-actin independently localizes throughout the cortex. These data suggest that independent isoform-specific actin arrays perform different functions required for the successful completion of cytokinesis.

## Results

### Anillin enhances RhoA effect on DIAPH3 DID–DAD interaction

As anillin binds to the DID region of DIAPH3^17^, we reasoned that anillin could participate in the localized activation of DIAPH3-dependent actin nucleation activity, specifically at the cleavage furrow. We confirmed that the DIAPH3 DID interacts with the first 151 amino acids of anillin^17^ (Supplementary Fig. 1a). Next we reconstituted auto-inhibited DIAPH3 by immobilizing maltose binding protein (MBP) fused to DIAPH3-NT, which contains the G-protein binding domain (GBD) and DID, then incubating it with GST-DIAPH3-CT, which in turn contains the FH1, FH2 actin-nucleating domains, and DAD regions (Fig. 1). The DIAPH3-NT + CT immobilized complexes were then incubated in the presence or absence of GST-RhoA and 6× His-tagged anillin (1–151), and the amount of GST-DIAPH3-CT remaining immobilized on beads was determined by immunoblotting (Fig. 1b). Equimolar concentrations of anillin did not significantly reduce the amount of DIAPH3-CT remaining immobilized on DIAPH3-NT beads compared to control (18 ± 0.03% and 8 ± 0.02% reduction in binding respectively; no statistically significant differences compared to the control *p* = 0.34, *n* = 3). Indeed, we detected no stable binding of anillin to the DIAPH3-NT + CT complex (Fig. 1d), indicating that anillin alone is not sufficient to relieve the DIAPH3 auto-inhibited state. In contrast, equimolar concentrations of activated RhoA reduced the amount of co-purifying DIAPH3-CT complex by 49 ± 0.03%, consistent with its observed ability to partially activate formins^21,22^. Strikingly, however, the addition of equimolar amounts of both anillin and activated RhoA reduced DIAPH3-CT levels immobilized on the DIAPH3-NT beads by 92 ± 0.05%, indicating that anillin enhances the RhoA-mediated release of the DIAPH3-DAD domain from the DID domain (Fig. 1b). Anillin binding to the DIAPH3-NT + CT complex only occurred in the presence of RhoA, whose own binding to the complex was enhanced by the presence of anillin (Fig. 1c, d). These data suggest that full DIAPH3 activation is potentiated by anillin in a mechanism, whereby RhoA binds to auto-inhibited DIAPH3 first followed by anillin binding.

### Anillin enhances DIAPH3 actin nucleation activity

**Fig. 2:**
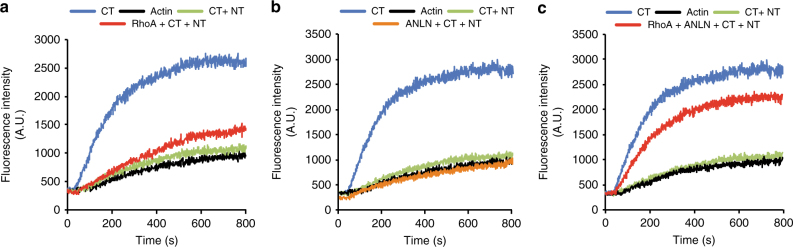
Anillin (ANLN) enhances RhoA-mediated activation of DIAPH3 actin nucleation activity. Pyrene-labeled actin polymerization assays comparing the relative actin polymerization activity of GST-DIAPH3-CT and a complex of GST-DIAPH3-CT + MBP-DIAPH3-NT in the presence of **a** GST-RhoA, **b** 6× His-anillin_(1–151)_, and **c** GST-RhoA and 6× His-anillin_(1–151)_

**Fig. 3:**
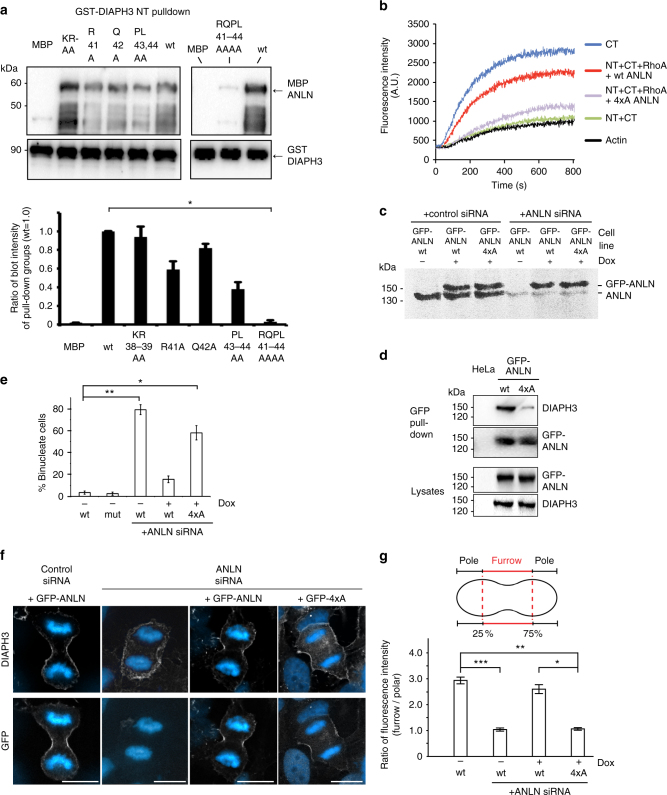
A direct interaction between anillin and DIAPH3 enhances RhoA-mediated activation of DIAPH3 actin nucleation activity and the successful completion of cytokinesis. **a** MBP-anillin_(1–151)_, mutants, and wild type were incubated with GST-DIAPH3-NT, then the GST fusion proteins re-isolated on glutathione agarose beads and immunoblotted to detect and quantify the relative amount of co-purifying MBP-anillin mutants and wild type. *n* = 3. Error bars indicate ± s.e.m. *p = 0.0072, Student’s *t* test, comparing wt to RQPL 41–44 AAAA. **b** Pyrene-labeled actin nucleation assays in the presence of DIAPH3-CT-NT, GST-RhoA, and either 6× His-anillin wild type (1–151) or 6× His-anillin 4xA (41-RQPL-44 to AAAA). **c** Lysates from stable HeLa cell lines with a single integrated cDNA encoding GFP-anillin-wt (GFP-ANLN) or GFP-anillin 41-RQPL-44 to AAAA (GFP-4xA) under the control of the tet regulator were probed with an anti-anillin antibody. GFP fusion protein expression was induced by adding 1 μg/ml doxycyclin (Dox). **d** GFP-anillin-wt (GFP-ANLN) and GFP-anillin-41-RQPL-44 to AAAA (GFP-4xA) were isolated from stable cell lines using a GFP-TRAP and co-purifying DIAPH3 detected by immunoblotting. **e** The number of binucleate cells, an indicator of cytokinetic failure, determined in the presence and absence of endogenous anillin, GFP-ANLN, or GFP-4xA. At least 100 cells were analyzed in each condition and in three experimental repeats. Error bars indicate ± s.e.m. *p = 0.002, **p = 0.007, Student’s *t* test. **f** DIAPH3 localization in HeLa cells stably expressing either GFP-ANLN or GFP-4xA anillin treated with control or anillin siRNA. Blue is DAPI staining. Scale bar is 10 μm. **g** Quantitation of endogenous DIAPH3 localization in the furrow region of a cell relative to the pole region of the cell. At least 100 cells were analyzed in each condition and in three experimental repeats. Error bars indicate ± s.e.m. *p = 0.007, **p = 0.002 and ***p = 0.001, Student’s *t* test

We next determined if anillin has a role in the actin nucleation activity of DIAPH3. By itself, the DIAPH3-CT domain induced efficient actin polymerization, whereas GST, MBP, DIAPH3-NT alone or the auto-inhibited DIAPH3-NT + CT complex did not (Fig. 2a; Supplementary Fig. 2a). Addition of activated RhoA to the DIAPH3-NT + CT complex caused a modest increase in actin polymerization compared to the complex alone (Fig. 2a; Supplementary Fig. 2d), consistent with RhoA’s effect on the activity of other formins^21,22^. Addition of anillin alone had no stimulatory effect (Fig. 2b; Supplementary Fig. 2c). In contrast, and consistent with our biochemical data, the addition of both anillin and activated RhoA to the DIAPH3-NT + CT complex dramatically stimulated actin polymerization, nearly to the level of the constitutively active DIAPH3-CT (Fig. 2c; Supplementary Fig. 2d). To determine if anillin has a direct role in activating DIAPH3, we generated a mutant form of anillin in the conserved N2 region, RQPL_41–44_ to 4xA (Supplementary Fig. 1b), that does not bind to DIAPH3 (Fig. 3a), but does bind to CD2AP and importin β2 that interact with anillin regions immediately surrounding RQPL^23,24^ (Supplementary Fig. 1c–e). Consistent with a role for anillin in DIAPH3 function, the anillin 4xA mutant failed to stimulate actin polymerization above the level of activated RhoA alone (Fig. 3b).

### The anillin–DIAPH3 interaction is required for cytokinesis

We next assessed the role of the anillin–DIAPH3 interaction during cytokinesis in vivo by generating stable cell lines expressing wt GFP-anillin (ANLN)^25^ or mutant GFP-anillin-RQPL_41–44_-AAAA (4xA) under the control of a tet repressor. The expression of GFP-anillins at levels similar to endogenous anillin was achieved by the addition of doxycycline (Dox) to the growth medium (Fig. 3c). As expected, endogenous DIAPH3 co-purified with GFP-ANLN isolated on GFP-TRAP beads. In contrast, very little DIAPH3 co-purified with GFP-4xA (Fig. 3d). Successful completion of cytokinesis was monitored by quantifying the number of binucleate cells, a hallmark of cytokinetic failure, in the presence and absence of ANLN and 4xA. Using siRNA directed against the 3′ UTR of endogenous anillin, we developed conditions to replace endogenous anillin with ectopically expressed ANLN or the 4xA (Fig. 3c). As expected, depletion of endogenous anillin resulted in a 20× increase in binucleate cells (79.6 ± 4.5% binucleate cells compared to 4.0 ± 0.5% in wild type, *p* = 0.002, Fig. 3e) and the loss of DIAPH3 recruitment to the cytokinetic ring (Fig. 3f, g). In contrast, expression of ANLN rescued both the binucleate phenotype (14.3 ± 3% binucleates) and DIAPH3 localization. Notably, 4xA expression neither rescued the cytokinesis defects (57 ± 6.5% binucleates, Fig. 3e *p* = 0.007 compared to the wild type) nor restored DIAPH3 enrichment at the furrow (Fig. 3f, g). Depletion of DIAPH3 has no detectable effect on anillin recruitment to the furrow (Supplementary Fig. 3).

**Fig. 4:**
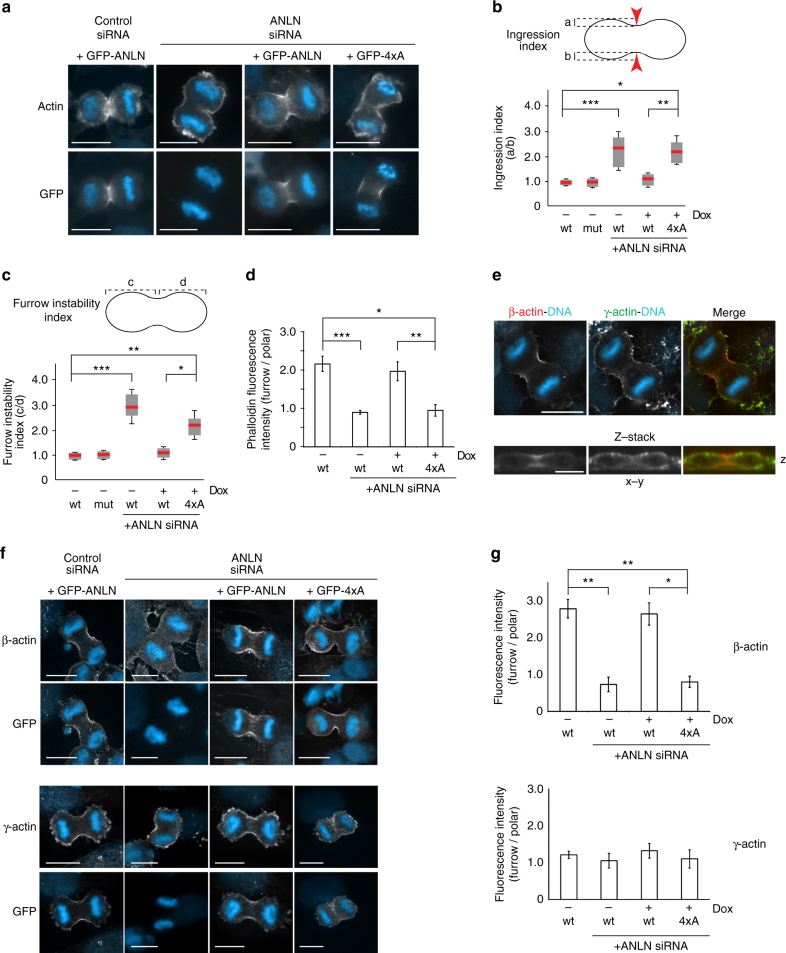
The anillin–DIAPH3 interaction is required for the early stages of furrow organization and β-actin polymerization at the cleavage furrow. **a** HeLa cells in anaphase stained with DAPI (blue) to mark chromosomes and phalloidin (white) to mark F-actin and the cell cortex. **b** Comparative furrow ingression indices, a measure of the degree of ingression of the furrow on each side of the cell where 1 = symmetrical ingression, in the presence and absence of endogenous anillin, GFP-ANLN, and GFP-4xA. Inter-chromosomal mass distance 8 μm + 0.1. Red bar = average, gray box = 25–75 percentile, and whiskers indicate data extremes. At least 100 cells were analyzed in each of three experimental repeats. *p = 0.013, **p = 0.003, ***p = 0.0025, Student’s *t* test. **c** Comparative furrow instability indices, a measure of furrow position, where 1 = furrow ingression in the middle of the cell in the presence and absence of endogenous anillin, GFP-ANLN, and GFP-4xA. Red bar = average, gray box = 25–75 percentile, and whiskers indicate data extremes. At least 100 cells were analyzed in each of three experimental repeats. *p = 0.004, **p = 0.003, ***p = 0.002, Student’s *t* test. **d** Comparative distribution of F-actin at the cell cortex in different conditions. At least 100 cells were analyzed in three experimental repeats. Error bars indicate ± s.e.m. *p = 0.006, **p = 0.005, ***p = 0.002, Student’s *t* test. **e** HeLa cells in anaphase stained with antibodies specifically recognizing β- and γ-actin during early anaphase. **f** Localization of β- and γ-actin in HeLa cells in the presence and absence of endogenous anillin, GFP-ANLN, and GFP-4xA. **g** Relative distribution of β- or γ-actin around the cell cortex. At least 100 cells were analyzed in each of three experimental repeats. Error bars indicate ± s.e.m. *p = 0.004, **p = 0.002, Student’s *t* test. All scale bars are 10 μm

To determine when cytokinesis became disrupted in cells where anillin and DIAPH3 did not interact, we performed timelapse imaging of cytokinesis in wild-type and mutant cells. In cells expressing ANLN, furrows formed in the middle of the cell and ingressed equally on all sides (Supplementary Movies 1, 2). Consistent with this data, in fixed cells expressing endogenous anillin and GFP-ANLN, the depth of cytokinetic furrows ingression was equidistant on each side of the equator and equidistant from either pole (ingression index, 0.98 ± 0.03 and 1 ± 0.1, respectively, furrow instability index 1.02 ± 0.02 and 1.12 ± 0.15, respectively, where 1 denotes symmetry, Fig. 4a–c). In contrast, cells expressing only the 4xA mutant exhibited disorganized and mis-positioned furrow ingression by live imaging (Supplementary Movie 3). Likewise, asymmetric furrows were also observed in fixed cells lacking endogenous anillin or expressing only 4xA (ingression index, 2.31 ± 0.25 and 2.19 ± 0.2, respectively, furrow instability index 2.95 ± 0.33 and 2.17 ± 0.3, respectively, Fig. 4a–c). Depletion of DIAPH3 by small interfering RNA (siRNA) phenocopied the 4xA anillin mutant effect on the ingression and furrow stability index (Supplementary Fig 3f, g).

Consistent with a role for anillin in DIAPH3 recruitment and function at the cytokinetic furrow, actin filament organization was markedly different between cells expressing ANLN vs. 4xA. In ANLN expressing cytokinetic cells, phalloidin labeled the entire cell cortex, with increased concentrations at the cleavage furrow (phalloidin distribution furrow:pole = 2.15 ± 0.26, Fig. 4a, d). In contrast, in siRNA–anillin depleted cells and cells expressing only 4xA, phalloidin staining was more uniform around the plasma membrane and failed to concentrate at the furrow (furrow:pole = 0.84 ± 0.02 and 0.92 ± 0.29, respectively; *p* = 0.002 compared to *p* = 0.006 in the wild-type cells expressing ANLN). Likewise, depletion of DIAPH3 phenocopied the 4xA anillin mutant in its failure to concentrate actin at the furrow (Supplementary Fig. 3d, e). These data suggest that anillin promotes furrow stability and successful cytokinesis at least in part by enhancing actin accumulation at the cytokinetic furrow through the recruitment and activation of DIAPH3 at the furrow.

### Anillin–DIAPH3 is required for β-actin at the furrow

The two ubiquitously expressed actin isoforms, β- and γ-, are highly conserved in vertebrates, and differ by only four biochemically similar residues at the N-terminus (Supplementary Fig. 4a). Although these sequence differences are very conservative, previous studies suggest that the actin isoforms may have distinct functions^26–30^. We therefore analyzed the distribution of β- and γ-actin during cytokinesis using isoform-specific antibodies^27^. During anaphase, γ-actin was uniformly distributed around the cortex of the cell (furrow:pole = 1.22 ± 0.1, Fig. 4e–g). In contrast, β-actin was enriched at the cytokinetic furrow (furrow:pole = 2.76 ± 0.25), corroborating a previous study^27^. Strikingly, depletion of endogenous anillin did not affect γ-actin distribution around the cell cortex, but did prevent the accumulation of β-actin at the cytokinetic furrow (furrow:pole = 0.69 ± 0.2). Depletion of DIAPH3 had similar effects on actin isoform distribution in cytokinesis (Supplementary Fig. 5). Moreover, β-actin filament enrichment at the cytokinetic furrow was rescued by the expression of ANLN, but not 4xA (furrow:pole = 2.62 ± 0.4 and 0.78 ± 0.15, respectively, Fig. 4f, g). Thus, the production of β-actin filaments at the cytokinetic furrow is dependent on the anillin–DIAPH3 interaction.

### DIAPH3 nucleates β-actin filaments

The simplest interpretation of our data is that DIAPH3 is a β-actin-specific nucleator. Alternatively, the DIAPH3-dependent assembly of β-actin filaments could derive from additional conditions unique to the cytokinetic furrow and mitosis. To distinguish between these possibilities, we targeted the DIAPH3-CT containing the actin-nucleating FH2 domain to the surface of mitochondria by fusing it to the mitochondrial-targeting region of TOM20 and GFP^31^. In wild-type cells, neither β- nor γ-actin was observed on mitochondria (Supplementary Fig. 6). In contrast, cells expressing the DIAPH3-CT-TOM20-GFP fusion protein accumulated β-actin, but not γ-actin, on and around mitochondria, indicating that the ability of DIAPH3 to nucleate β-actin filaments is not restricted to the cytokinetic furrow.

**Fig. 5:**
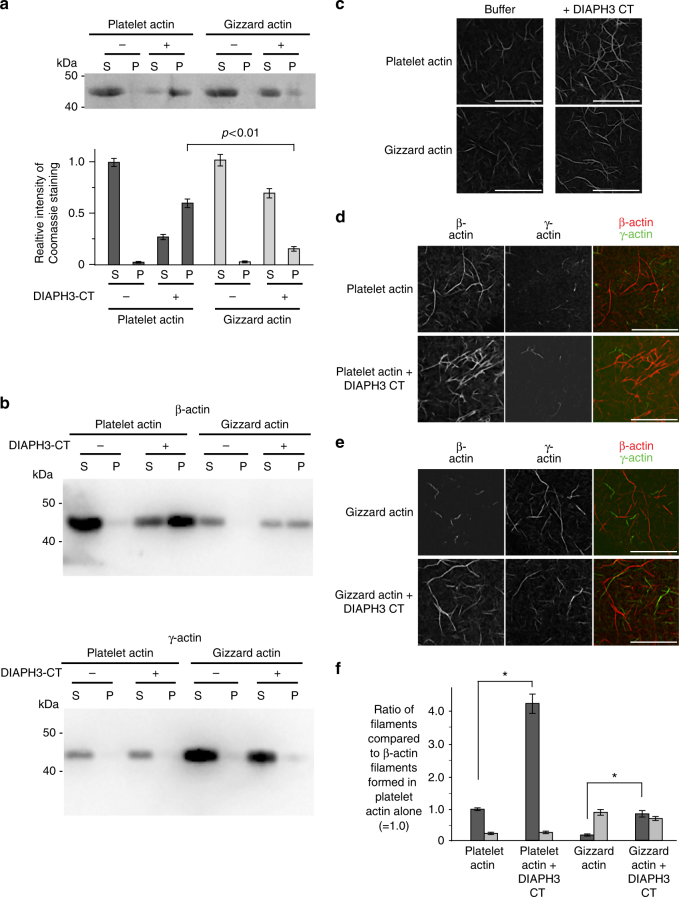
DIAPH3 preferentially nucleates β-actin filaments over γ-actin filaments. **a** Actin pelleting assay in the presence and absence of GST-DIAPH3-CT using actin isolated from human platelets or chicken gizzard. The polymerization reaction was carried out for 30 min. P = pellet fraction, polymerized actin. S = actin in the supernatant, unpolymerized actin. *n* = 3. Error bars indicate ± s.e.m. **b** Immunoblots of the actin pelleting assay in A, probed with β- and γ-actin-specific antibodies. P = pellet fraction, polymerized actin. S = actin in the supernatant, unpolymerized actin. **c** On glass actin nucleation assay, where actin filaments are fixed and visualized by phalloidin staining. **d** Actin isolated from human platelets nucleated on glass by GST-DIAPH3-CT, fixed, and stained with β- and γ-actin-specific antibodies. **e** Actin isolated from chicken gizzard nucleated on glass by GST-DIAPH3-CT, fixed, and stained with β- and γ-actin-specific antibodies. **f** Quantitation of the number of filaments per field relative to the number of filaments generated upon incubation of human platelet actin in buffer. *n* = 3. Error bars indicate ± s.e.m. *p < 0.01, Student’s *t* test. All scale bars are 10 μm

We next determined whether DIAPH3 preferentially nucleates β-actin vs. γ-actin filaments by performing in vitro actin polymerization assays using actin purified from different sources. Human platelet actin is ~83% β-actin and 17% γ-actin, whereas chicken gizzard actin is ~76% γ-actin and 24% β-actin (Supplementary Fig. 4b, c). The actin from the different sources exhibited no significant differences in their ability to generate filaments in vitro (Supplementary Fig. 7). We then incubated DIAPH3-CT with the different actins, harvested the actin filaments by ultracentrifugation and quantified the abundance of actin filaments by Coomassie blue staining of sodium dodecyl sulfate–polyacrylamide gel electrophoresis (SDS–PAGE) gels (Fig. 5a). DIAPH3-CT generated ~9-fold more filaments when incubated with the β-actin-rich human platelet actin compared with the γ-actin-rich gizzard (polymerized:unpolymerized = 2.31 platelet, 0.26 gizzard; *p* = 0.003). This difference in actin polymerization resulted from a strong substrate bias: DIAPH3-CT preferentially promoted the assembly of β-actin vs. γ-actin filaments, as judged by both Western blotting using isoform-specific antibodies (Fig. 5b) and compellingly by the isoform-specific immunostaining of actin filaments nucleated on glass coverslips (Fig. 5c–f). When DIAPH3-CT was incubated with either β-rich platelet or γ-rich gizzard actin, the production of β-actin filaments was stimulated four-fold (4.2 and 4, respectively) as judged by the increased number of β-actin filaments (*p* = 0.007 compared to *p* = 0.006 in the control groups without DIAPH3-CT), whereas there was no change in the number of γ-actin filaments (Fig. 5d–f). In all cases, it is noteworthy that the actin filaments generated were homopolymeric, and contained either β-actin or γ-actin: no mixed isoform filaments were observed (Fig. 5d, e). Taken together, our data indicate that in vitro, the formin DIAPH3 preferentially nucleates homopolymeric β-actin filaments compared with γ-actin filaments. We propose that this differential nucleation activity accounts at least in part for the enrichment of β-actin filaments at the cytokinetic furrow.

## Discussion

Here we show that β-actin filaments are predominantly generated at the cytokinetic furrow during anaphase, where they are specifically required to organize and stabilize the ingressing furrow. β-actin filament generation requires the formin DIAPH3, which preferentially nucleates β-actin but not γ-actin filaments. Full activation of DIAPH3 requires both an activator RhoA and an enhancer, anillin, which are both enriched at the cytokinetic furrow and thus provide exquisite spatio-temporal control of β-actin filament production between segregating masses of chromosomes.

The diaphanous branch of the formin family exist in an auto-inhibited state mediated by an intramolecular interaction between the DAD and DID domains (Fig. 1a). Assays using either fragments of DIAPH1 or full-length DIAPH1 demonstrated that the binding of the small G-protein, RhoA, to DIAPH1 only partially relieves the auto-inhibition of actin polymerization activity^21,22^, suggesting a requirement for additional factors. In the case of DIAPH3, we have now identified one such enhancer, anillin that binds to DIAPH3 after RhoA binding to strongly enhance the actin-nucleating activity of DIAPH3. How RhoA and anillin combine mechanistically to activate DIAPH3 is unknown. Rho binding releases the auto-inhibitory interaction between DID and DAD due to the partially overlapping binding sites of RhoA and DAD^32,33^. This could then allow anillin access to the DID, preventing the re-association of DAD to DID. This model would be consistent with previous in vitro binding assays^17^. Alternatively, Rho could induce a conformational change in DIAPH3 that reveals an anillin-binding site, the combined effect being to more efficiently release DAD from DID to fully activate DIAPH3.

Interestingly, our in vitro data show that combined Rho and anillin binding, although significantly increasing DIAPH3 activity, did not activate DIAPH3 to the same level as the FH1-FH2 domain alone. This could simply be due to the use of protein fragments or reflect a requirement for additional activators. For example, the Fli-1 protein can enhance Rho-mediated activation of both the DIAPH1 and DAAM1 formins by binding to DAD^34^. By utilizing multiple factors to fully activate the actin-nucleating activity of a formin, the cell can tightly regulate cellular actin production. Furthermore, if each of these components is subject to strict spatio-temporal regulation within the cell, as is the case with Rho and anillin^35–37^, the cell can exert precise control over when, where, and at least for DIAPH3, what type of actin filaments are generated.

The existence of multiple actin isoforms, encoded by different genes, has long been known, though ascribing distinct function to these highly conserved yet distinct isoforms has proven challenging^38^. In non-muscle cells, cytoplasmic β-and γ-actin are ubiquitously expressed. These isoforms are highly conserved between each other and across species. β- and γ-actin differ by only four conservative amino acids changes at the extreme N-terminus of the protein (within the first 10 amino acids) (Supplementary Fig. 4). Despite this nearly identical sequence, β- and γ-actin are associated with different functions in vivo including roles at tight junctions and in cell migration^26–30^. Our data support the existence of specific roles for distinct actin isoforms and identifies DIAPH3 as a β-actin rather than a γ-actin nucleator. How DIAPH3 exerts this isoform preference is unclear, as most of the formin–actin interaction sites observed in the yeast formin Bni1p-actin crystal structure occur in regions that are conserved between β- and γ-actin^39^. While the divergent N-terminus of actin is not visible in the crystal and may underlie the functional distinction between actins, it is possible that differential post-translation modifications between actin isoforms could account also for DIAPH3 preference for β- over γ-actin. While many post-translational modifications of actin have been reported, few have been correlated with a specific function^40^. Oxidation of methionine residues within the N-terminal region of actin by MICAL1 is however required for actin depolymerization in the late stages of cytokinesis^41^. One striking difference between β- and γ-actin is the spectrum of post-translation modifications that occur at the extreme N-terminus. All actin isoforms are processed post-translationally such that the amino terminal methionine is cleaved and the first acidic amino acid at position 2 (D2 vs. E2 in β- vs. γ-actin) is acetylated. Uniquely β-actin can undergo a further modification, N-terminal arginylation that occurs in about 40% of cellular β-actin^26,42^. This seems unlikely to account for the DIAPH3 preference for β- vs. γ-actin nucleation in vitro, however, as we detected no arginylated peptides in β-actin from either platelet or gizzard. While β-actin contains three aspartic acid residues (aa 2–4) vs. three glutamic acid residues (aa 2–4) in γ-actin, the N-terminus of α-actin, whose expression is restricted largely to muscle, is a hybrid of β- and γ-actin containing a mix of aspartates and glutamates (Supplementary Fig. 4). In vitro DIAPH3 can polymerize pyrene-labeled α-actin, suggesting that there is something peculiar to γ-actin that prevents nucleation by DIAPH3. It is tempting to speculate that as with histones^43^ and tubulin^44^, there is an actin “code” derived from a varied combination of N-terminal sequences and post-translational modifications that define the when, where, and function of distinct arrays of the actin cytoskeleton.

Our in vivo data suggest that β-actin filament generation at the cytokinetic furrow is required for stable furrow ingression and successful cytokinesis. In turn this suggests that β-actin filaments have some intrinsic property that is required at the furrow that γ-actin filaments do not offer. The nature of this property is unclear. In vitro β-actin exhibits higher polymerization rates than γ-actin in the absence of a nucleator^45^. If furrow ingression and stability required faster generation of actin filaments, this could explain the need for β-actin filaments at the furrow vs. γ-actin. An alternative model, although not mutually exclusive, is that different factors bind to β- and γ-actin filaments. There is precedent for this as ezrin^46^ and different myosins^47^ have different affinities for different actin isoforms. Consequently, the different actin isoform arrays would have different biochemical characteristics enabling them to perform distinct cellular functions. It would be vital then to tightly regulate when and where isotype-specific actin filaments are generated. Previous studies using in vitro pyrene-labeled actin polymerization assays suggested that mixed β-actin-γ-actin polymers form^45^. In contrast, our microscopy-based assays could not detect any mixed polymer filaments: filaments were either β-actin or γ-actin. Interestingly this was also true for polymers generated in the absence of added DIAPH3. The ability to form isoform-specific filaments in vivo would then allow a separation of biochemical activities within the cell on the different actin arrays.

How actin is organized within the furrow and the source of actin there has been intensely debated. Actin arrays at both the furrow and the poles are required for cytokinesis^3^. Evidence suggests that at least two processes are at play: the cortical flow of actin from the poles and the de novo production of actin at the furrow^4–7^. Our data raise the possibility that these different processes involve different actin isoforms: β-actin in de novo actin production at the furrow and γ-actin in cortical flow. How these two arrays combine to allow successful cytokinesis, whether it be through different organizational states or the differential recruitment of factors to generate regions with distinct biochemical activities or a combination of both remains to be determined.

Previous models implicated regulated β-actin messenger RNA (mRNA) localization in dictating, where β-actin arrays were formed within the cell^48–50^. Our study demonstrates an additional tier of regulation, that of targeted localization and activation of actin nucleators. DIAPH3 targeting to the cytokinetic furrow is dependent upon anillin binding, which itself is targeted to the furrow through an interaction with the lipid PI(4,5)P_2_^37,51^ that becomes enriched in the furrow. Rho-GTP also concentrates in the furrow^36^. Combined Rho and anillin would ensure localized targeting and activation of DIAPH3 to generate β-actin filaments at the furrow that are required to drive stable furrow ingression and successful cytokinesis.

## Methods

### cDNA cloning

Complimentary DNAs (cDNAs) fragments of human anillin (MGC 87306), DIAPH3 (Addgene plasmid 25407) were amplified by PCR using the i-Max II DNA polymerase (Froggalab) using oligonucleotide primers (Integrated DNA Technologies) listed in Supplementary Table 1. PCR fragments were cloned using the TOPO Gateway system (Life Technologies) being first cloned into the entry plasmid vector pCR8/GW/TOPO, then moved into the desired destination vectors: pDEST 15 to generate GST fusion protein, pDEST 17 (Life Technologies) to generate 6× His-tagged proteins or pKM596 (Addgene plasmid 8837) to generate MBP fusion proteins. Mutations within the anillin cDNA, were generated using oligonucleotide primers, and are listed in Supplementary Table 1.

### Protein expression and purification

Recombinant GST and 6× His-tagged proteins proteins were expressed and purified from BL21 *Escherichia coli* cells and MBP fusion proteins expressed and purified from ER2 *E*. *coli* (New England Biolabs)^23^. In brief, cells were grown in LB media at 37 °C to an optical density of 0.6 at *A*_600_. Recombinant protein expression was induced by the addition of 1 mM isopropyl β-d-1-thiogalactopyranoside (IPTG) and further incubated at 16 °C overnight. Cells were harvested by centrifugation and stored in −80 °C.

To purify GST fusion proteins, *E*. *coli* cells were resuspended in 25 mM HEPES, pH 7.5, 250 mM NaCl, 100 mM KCl, 0.5 mM β-Mercaptoethanol, 1 mM PMSF, and lysed by sonication. The lysates were cleared by centrifugation at 10,000×*g* for 30 min at 4 °C and applied to glutathione beads (Fermentas). The glutathione beads were washed with 10 column volumes of column buffer (CB: 25 mM HEPES, pH 7.5, 250 mM NaCl, 100 mM KCl, 0.5 mM β-Mercaptoethanol, 1 mM PMSF, 0.1% (v/v) Triton X-100), and the GST fusion proteins were eluted in CB containing 10 mM glutathione.

To purify MBP fusion proteins, *E*. *coli* cells were harvested and lysed as above. The lysates were applied to amylose resin (New England Biolabs). The resin was washed with 10 column volumes of CB and the MBP fusion proteins were eluted in CB containing 10 mM maltose.

To purify 6× His fusion proteins, *E*. *coli* cells were resuspended in 25 mM HEPES, pH 7.5, 500 mM NaCl, 5% (v/v) glycerol, 5 mM imidazole, 0.5 mM β-Mercaptoethanol, 1 mM PMSF, and lysed by sonication. The lysates were cleared by centrifugation as above then applied to nickel-Sepharose beads (Amersham Biosciences). The beads were washed with 10 column volumes of 6× His column buffer (HCB: 25 mM HEPES, pH 7.5, 500 mM NaCl, 5% (v/v) glycerol, 5 mM imidazole, 0.5 mM β-Mercaptoethanol, 1 mM PMSF, 0.1% (v/v) Triton X-100. The 6× His fusion proteins were eluted in HCB containing 500 mM imidazole.

Eluted proteins were dialyzed into 10 mM HEPES, pH 7.6, 100 mM KCl, 2 mM MgCl_2_, 50 mM sucrose overnight at 4 °C and concentrated using Millipore Ultrafree spin columns (Milipore, Ireland) with a 10-kDa cutoff. Proteins were then aliquoted, flash-frozen in N_2_(l), and stored at −80 °C.

To generate GTP- or GDP-loaded GST-RhoA for the in vitro binding and actin polymerization assays, purified GST-RhoA fusion proteins were added to 25 mM EDTA, 1 mM DTT, and GTP or GDP added to a 100× molar excess of the proteins. The reactions were incubated on ice for 40 min before adding MgCl_2_ to a final concentration of 50 mM. GTP- or GDP-loaded proteins were then dialyzed, concentrated, and stored as described above.

### In vitro binding assays

To map the anillin–DIAPH3 binding site, 0.05 nmol of MBP-NT or GST-CT was immobilized onto 20 μl glutathione agarose or amylose beads, respectively, in 100 μl of incubation buffer (IB: 50 mM Hepes pH 7.5, 50 mM NaCl, 1 mM EDTA, 5 mM MgCl_2_, 0.3% (v/v) Triton X-100, 1 mM β-Mercaptoethanol) and incubated for 1 h at 4 °C. The beads were washed in IB (all subsequent washes were done in this buffer unless otherwise noted) and blocked with 3% (w/v) BSA for 20 min. The resin was then washed and mixed with 0.02 nmol of different MBP anillin fragments and incubated for 2 h at 4 °C. Unbound protein was removed by washing the beads in IB, which were next re-isolated by centrifugation and boiled in SDS sample buffer then analyzed by Western blotting using an anti-MBP monoclonal antibody (E8032, New England Biolabs, 1:2000 dilution) to detect co-purifying anillin fragments.

To assess the role of anillin and RhoA in regulating the interaction between DIAPH3-NT and DIAPH3-CT, 0.05 nmol of MBP-DIAPH3-NT was immobilized onto 20 μl amylose resin in 100 μl IB as described above and incubated for 1 h at 4 °C. The resin was washed to remove unbound protein, then blocked with 3% (w/v) BSA for 20 min and further washing. About 0.05 nmol GST-DIAPH3-CT was then incubated with the resin for 2 h at 4 °C, followed by a wash step. 6× His-anillin and GST-RhoA Q63L were then added to the resin, incubated for 2 h then washed in IB to remove unbound protein. The beads were re-isolated by centrifugation, boiled in SDS sample buffer then analyzed by Western blotting using an in house rabbit anti-GST polyclonal antibody (1:1000 dilution) to detect co-purifying GST-DIAPH3-CT. To determine if anillin or Rho bound to the DIAPH3 CT-NT complex first, the same protocol was used except analysis was carried out with an anti-6× His polyclonal antibody (MP Biomedicals, 1:500 dilution) to detect co-purifying anillin and the homemade anti-GST polyclonal antibody to detect co-purifying Rho.

To determine if importin β2, CD2AP, or DIAPH3 competed for anillin binding, 0.05 nmol of MBP-ANLN (1–151) was immobilized onto 20 μl of amylose resin beads in 100 μl IB for 1 h at 4 °C. The resin was washed, blocked with 3% (w/v) BSA for 20 min and washed once more prior to the addition of 0.02 nmol of GST-NT. After 2 h incubation at 4 °C, the resin was washed with IB and different concentrations of 6× His-importin β2^23^ was added, followed by a 4 h incubation at 4 °C. The resin was washed with IB, then re-isolated by centrifugation and boiled in SDS sample buffer. The amount of co-purifying GST-DIAPH3-NT was determined by SDS–PAGE detected followed by probing a Western blotting with an anti-GST polyclonal antibody.

To determine if CD2AP and DIAPH3 shared a common binding site on anillin, 0.05 nmol of MBP-ANLN (1–151) was immobilized onto 20 μl amylose resin, blocked by BSA and further incubated with GST-NT as described above. The resin was then washed with IB and different concentrations of GST-CD2AP^23^ added and incubated for 2 h at 4 °C. The resin was washed with IB, re-isolated by centrifugation and boiled in SDS sample buffer. The amount of co-purifying GST-DIAPH3-NT was determined by SDS–PAGE detected followed by probing a Western blotting with an anti-GST polyclonal antibody. Uncropped blots and gels are shown in Supplementary Figs. 8–11.

### Quantification of western blots

The PVDF membrane of Western blots was developed by chemiluminescent solution (Life Technologies) for 5 min at room temperature and visualized using a BioRad MP Imager (Bio-Rad, Canada). The intensities of individual bands on the blots were measured using ImageLab software (Bio-Rad Inc.). To determine the relative binding in the in vitro competition assays (Fig. 1b; Supplementary Fig 3a, b), a control pulldown reaction of wild-type “bait” protein that bound to beads and prey protein was performed and run on each gel. The intensity of the of bait protein band that bound to beads and the band of prey protein that was pulldown were set as the control standard to 1. In the comparative reactions, where potential competitor proteins were added or changing concentrations of proteins were added, the band intensity of the bait protein in each reaction was first compared and the differences used to adjust the band intensity of the amount of co-purifying prey protein to allow comparison. The adjusted prey band intensities were then compared to the intensity of the prey in the control reaction. Each binding assay was repeated at least three times. Analogous strategies were used to compare direct in vitro binding assays in Fig. 2b. The relative intensity of different bands were compared using a Student’s *t* test.

### Generation of stable cell lines

Mutants were generated by PCR using oligonucleotides that encoded the relevant mutation (Supplementary Table 1). A full-length anillin cDNA including the RQPL (41–44)-AAA(4xA) mutation was amplified by PCR and cloned into a modified pcDNA 5 FRT/TO vector downstream of GFP^25^ using the InFusion system (Promega). Stable HeLa cells lines with regulated expression of the mutant anillin under control of the Tet repressor were generated using the Flp-In system (Life technologies) using HeLa cells that contained a single FRT site^25,52^. The resulting cell lines were cultured in DMEM (Sigma) supplemented with 10% fetal bovine serum (Life Technologies), 5 µg/ml blasticidin (Bioshop), and 200 µg/ml hygromycin (Bioshop) in a 5% CO_2_ atmosphere at 37 °C. GFP fusion protein expression was induced by incubating stable HeLa cell lines with 1 μg/ml Dox for 24 h and expression of the GFP fusion detected by Western blotting with an anti-anillin antibody (sc-67327, Santa Cruz Biotechnology, 1:200 dilution).

### GFP-ANLN TRAP assay

To assess the comparative binding of anillin vs. anillin RQPL (41–44)-AAAA(4xA) to DIAPH3 in vivo, stable HeLa cell lines expressing eGFP-anillin and eGFP-anillin RQPL (41–44)-AAAA were grown to 75% confluency in 60 mm dishes and eGFP-fusion protein expression was induced by the addition of 1 µg/ml Dox for 24 h prior to analysis. Cells were lysed in 200 μl ice-cold non-denaturing lysis buffer (1% (w/v) Triton X-100, 150 mM NaCl, 10 mM Na_2_HPO_4_ pH 7.2, 2 mM EDTA, 50 mM NaF) with 1% (v/v) Protease Inhibitor Cocktail (Roche). Lysates were then adjusted to 1 ml with wash buffer (150 mM NaCl, 10 mM Na_2_HPO_4_ pH 7.2, 0.5 mM EDTA) and incubated with 20 μl GFP-Trap beads (Chromotek) overnight at 4 °C with rotation. GFP-Trap beads were then washed with wash buffer, boiled in SDS sample buffer and analyzed by Western blotting with mouse anti-DIAPH3 antibody (4D5 sc-293288 Santa Cruz Biotechnology, 1:500 dilution).

### Pyrene-labeled actin polymerization assays

To determine the actin polymerization activity of DIAPH3, 1 mg lyophilized pyrene-labeled or unlabeled actin (cytoskeleton) was resuspended in 50 µl H_2_O at 4 °C, then 150 µl G-buffer (monomer actin buffer: 2 mM Tris pH 8.0, 0.2 mM CaCl_2_, 0.2 mM ATP, 0.5 mM β-Mercaptoethanol) was added to make an actin stock (58 µM) and incubated on ice for 2 h. For individual assays, the actin stock was further diluted in G-buffer and the pyrene-labeled actin was mixed with unlabeled actin in a 15%: 85% ratio to make a final concentration of 2 µM total actin. Freshly purified proteins were added to actin at a concentration of 2.5 nM and incubated for 5–10 min at room temperature. The reaction was initiated by adding polymerization buffer (25 mM Tris pH 7.0, 50 mM KCl, 2 mM MgCl_2_, 0.1 mM ATP). The increase in fluorescence intensity was monitored in a PTI fluorimeter with excitation at 365 nm and emission at 386 nm as ref. ^53^. The initial time point was set to when the polymerization buffer was added and the reaction was monitored for 800 s. The *t*_(1/2)_ was defined as the time point when half amount of total actin monomers polymerized to filaments^54^. Actin polymerization rates at *t*_(1/2)_ were calculated using the same methods as previously described^54^. In brief, 10 data points at the minimum and the maximum intensities were chosen and used to calculate *I*_min_ (the average minimum intensity) and *I*_max_ (the average maximum intensity). The data point with intensities between 0.48 × (*I*_max_−*I*_min_) + *I*_min_ and 0.52 × (*I*_max_−*I*_min_) + *I*_min_ was chosen, fitted to a linear line and where the slope = *m*_(1/2)_ and the intercept = *b*_(1/2)_. Subsequently the *t*_(1/2)_ (time point when half amount of total actin monomers polymerize to filaments) was calculated as: *t*_(1/2)_ = (0.5 × (*I*_max_−*I*_min_) + *I*_min_ + *b*_(1/2)_)/*m*_(1/2)_, the AP *t*_(1/2)_ (actin polymerization rate at *t*_(1/2)_) as AP_*t*(1/2)_ = 1.88 × *m*_(1/2)_/(*I*_max_−*I*_min_).

### In vitro actin pelleting assays

To assess if DIAPH3 preferentially produces β-actin filaments rather than γ-actin filaments, 1 mg lyophilized platelet actin or gizzard actin (cytoskeleton) was first resuspended in 50 µl H_2_O, then 150 µl G-buffer was added to make an actin stock (58 µM) and incubated on ice for 2 h. Actin was then further diluted in G-buffer to 2 μM final and freshly purified DIAPH3 in 2 mM Tris pH 8.0, 0.5 mM β-Mercaptoethanol was added to the actin solution at a final concentration of 5 nM DIAPH3. The actin polymerization reaction was initiated by adding polymerization buffer and the mixture was incubated at 23 °C from 5 to 120 min depending on the condition of individual assays. Actin filaments were recovered by ultracentrifugation at 65,000 rpm for 20 min in a TLA 120.2 rotor applied in Beckman TL-100 Ultracentrifuge (Beckman Coulter, Inc.) Supernatants were carefully removed from the pellets and both supernatant and pellets were boiled with SDS sample buffer and analyzed by SDS–PAGE and Coomassie Blue staining or by Western blotting using anti β-actin or γ-actin antibodies (4C2 and 2A3 AbD Serotec, respectively, 1:400 dilution for IF, 1:100 dilution for WB)^27^. To determine the comparative amount of actin in soluble unpolymerized fraction (S) compared to the insoluble polymerized fraction (P), the Coomassie stained gels were scanned in a BioRad MP Imager (Bio-Rad, Canada) and the band intensities measured using ImageLab software (Bio-Rad Inc.). The intensity of the actin band in the platelet actin alone pelleting assay that remained in the solution (i.e., the soluble unpolymerized actin) was defined as the control and this intensity set to 1. The intensity of the bands in the different fractions and reaction conditions were then compared to the control as described above. Each actin pelleting assays was repeated at least three times.

### In vitro on glass actin polymerization assay

To visualize the production of actin filaments in the actin polymerization assays, the polymerization reactions were performed on 18 mm diameter glass coverslips pre-treated with sterile poly-lysine solution (0.5 mg/ml in 0.15 M borate buffer, pH 8.3 for 6 h followed by washing in sterile H_2_O and air-drying for another 2–4 h prior to use). Briefly, the actin polymerization assays was set up as described above and immediately after the initialization of polymerization, the reaction mixture was transferred to the coverslips and incubated at room temperature for 20 min. The solution was carefully removed from the coverslips and the coverslips were directly fixed using a 3.7% PFA solution followed by −20 °C methanol incubation for 15 min. The coverslips were then blocked by incubation with 3% BSA at room temperature for 1 h. The coverslips were next washed with PBS and incubated with anti-β- and γ-actin antibodies^27^ (AbD Serotec) at 4 °C overnight. The coverslips were washed with PBS and further incubated with secondary goat anti-mouse IgG1 (γ1) conjugated to Alexa 488, and goat anti-mouse IgG2b (γ2b) conjugated to Alexa 594 antibodies (Life Technologies, 1:1000 dilution) to visualize both β- and γ- actin filaments.

### siRNA rescue assays

HeLa cells were transfected with 40 nM double-strand anillin siRNA using Lipofectamin 2000 Reagent (Invitrogen)^25,37^. For rescue experiments, 16–24 h after siRNA treatment, cells were treated with 1 µg/ml Dox to induce the expression of eGFP-anillin transgene. siRNAs were obtained from Integrated DNA Technologies. The siRNA duplexes used in the assays were listed in Supplementary Table 2.

### Immunofluorescence and microscopy analysis

To visualize HeLa cells expressing eGFP-anillin, cells were fixed with 3.7% PFA at room temperature for 10 min, permeabilized for 10 min with 1% Triton X-100 in PBS then stained with 4′, 6-diamidino-2-phenylindole (DAPI) to visualize DNA. To visualize cellular β- or γ-actin structures, cells were fixed with pre-warmed 3.7% PFA for 30 min. Cells were washed three times with PBS, then post-fixed with methanol at −20 °C for 15 min. Cells were stained by anti-β actin or mouse anti-γ antibodies (AbD Serotec). Subsequently cells were washed three times with PBS; a secondary goat anti-mouse antibodies conjugated to Alexa 594 (Invitrogen) were used to visualize the cellular β- or γ-actin structures. Alternatively cells were stained with rhodamine-labeled phalloidin (Invitrogen, 1:1000 dilution) to visualize the total actin cytoskeleton. Coverslips were mounted on glass slides using Mowiol (Polyvinyl alcohol 4-88, Fluka). Cells were visualized using a Perkin Elmer UltraView spinning disk confocal scanner mounted on a Nikon TE2000-E with a ×60/1.4 NA oil-immersion objective lens and 1.515 immersion oil at room temperature. Images were acquired using METAMORPH software (Molecular Devices) driving an electron multiplying charge-coupled device (CCD) camera (ImagEM, Hammamatsu)^25^. *Z* sections (0.2 µm apart) were acquired to produce a stack that was then imported into AUTOQUANT X2 (Media Cybernetics) for deconvolution (10 iterations). Maximum projections and cross sections were performed using METAMORPH. Images were overlaid in PHOTOSHOP (Adobe), involving adjustments in brightness and contrast of images.

### Mitochondrial targeting assay

DIAPH3-CT (amino acids 501–1193) was cloned downstream of a Tom20Tm-GFP fusion (a gift from P. Kim Hospital for Sick Children, Toronto) using oligonucleotides listed in Supplementary Table 1. Plasmids encoding the Tom20Tm-GFP or the Tom20Tm-GFP-DIAPH3-CT were transfected into HeLa cells using Lipofectamin 2000 (Invitrogen) and fixed 24 h later in 3.7% PFA and probed with anti b- and g-actin antibodies (AbD Serotec).

### Data availability

The data that support the findings of this study are available within the paper and its Supplementary Information. Further details can be obtained from the corresponding author upon request.

## Electronic supplementary material

Supplementary Information

Description of Additional Supplementary Files

Supplementary Movie 1

Supplementary Movie 2

Supplementary Movie 3
