# Supplementary material for: Cytokinesis requires localized β-actin filament production by an actin isoform specific nucleator
Source: Nat Commun. 2017 Nov 16;8:1530. doi: 10.1038/s41467-017-01231-x (PMC5691081; doi:10.1038/s41467-017-01231-x)
Supplement: Supplementary file 1 — Supplementary Information [file 41467_2017_1231_MOESM1_ESM.pdf]

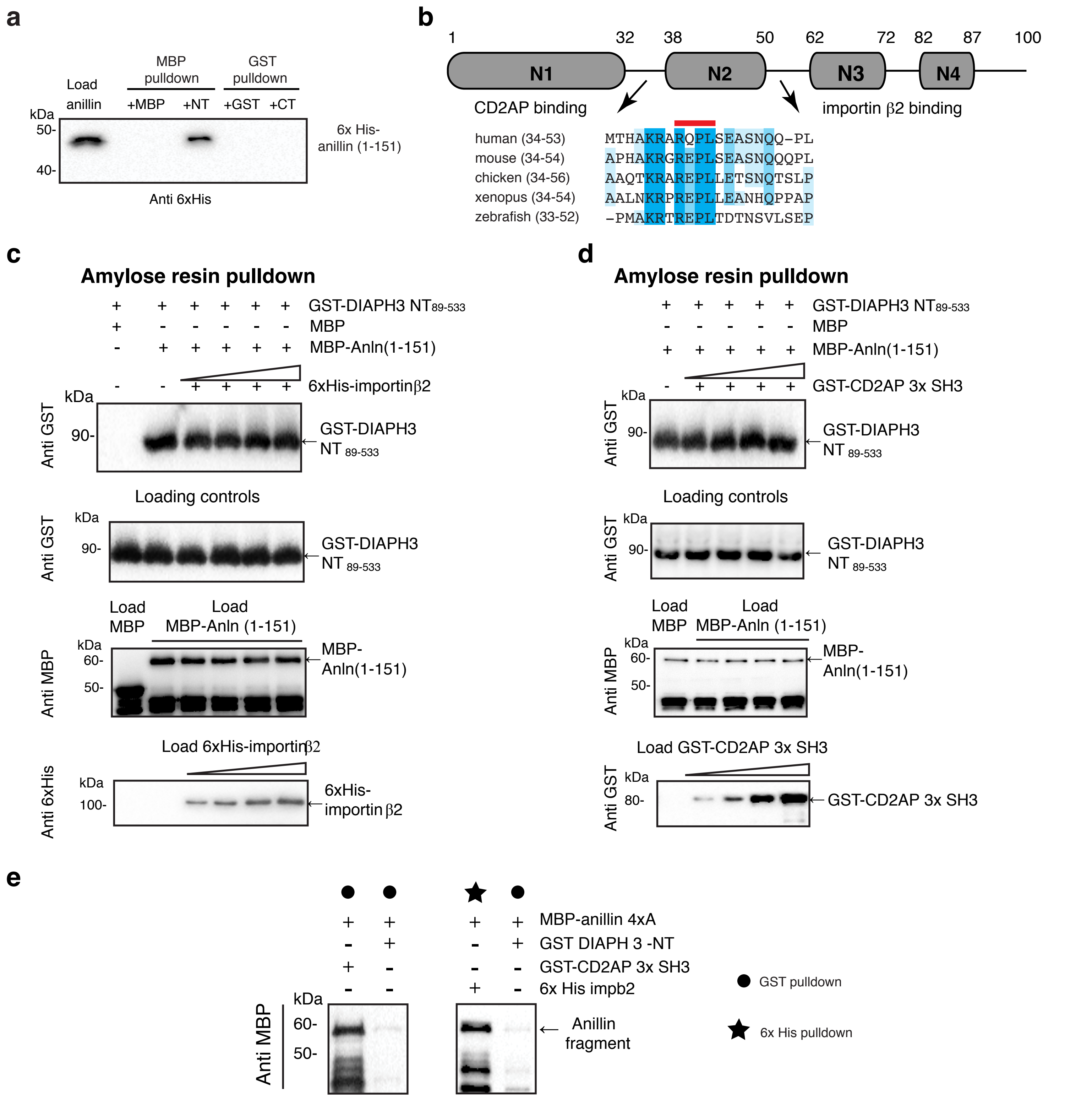


**Supplementary Figure 1.** DIAPH3 binds the N-terminal RQPL motif of anillin. **(a)** Recombinant 6x His Anillin(1-151) was incubated with GST-DIAPH3-CT or MBP-DIAPH3-NT. DIAPH3 fragments were re-isolated on glutathione agarose amylose resin beads respectively and co-purifying anillin detected by immunoblotting using an anti-6x His antibody. **(b)** Schematic diagram of the conserved regions within the first 100 amino acids of anillin in different vertebrate species with a detailed alignments of the N2 region below. Red bar indicates the 41-RQPL-44 sequence required for DIAPH3 binding. The darker the blue the greater the degree of conservation. **(c)** MBP-anillin(1-151) was incubated with GST-DIAPH3-NT and increasing concentrations of 6x His importin β2. MBP-anillin(1-151) was then re-isolated on amylose beads and co-purifying GST-DIAPH3-NT and 6x His importin β2 detected by immunoblotting. **(d)** MBP-anillin(1-151) was incubated with GST-DIAPH3-NT and increasing concentrations of GST-CD2AP. MBP-anillin(1-151) was then re-isolated on amylose beads and co-purifying GST-DIAPH3-NT and GST-CD2AP detected by immunoblotting. **(e)** GST-CD2AP-3x SH3 or 6x His importin β2 were incubated with MBP-anillin(1-151) wt and 41-RQPL-44 to AAAA (4xA). GST-CD2AP-3x SH3 was re-isolated on glutathione beads and 6x His importin β2 re-isolated on Ni-Sepharose and co-purifying MBP-anillin detected by immunoblotting.

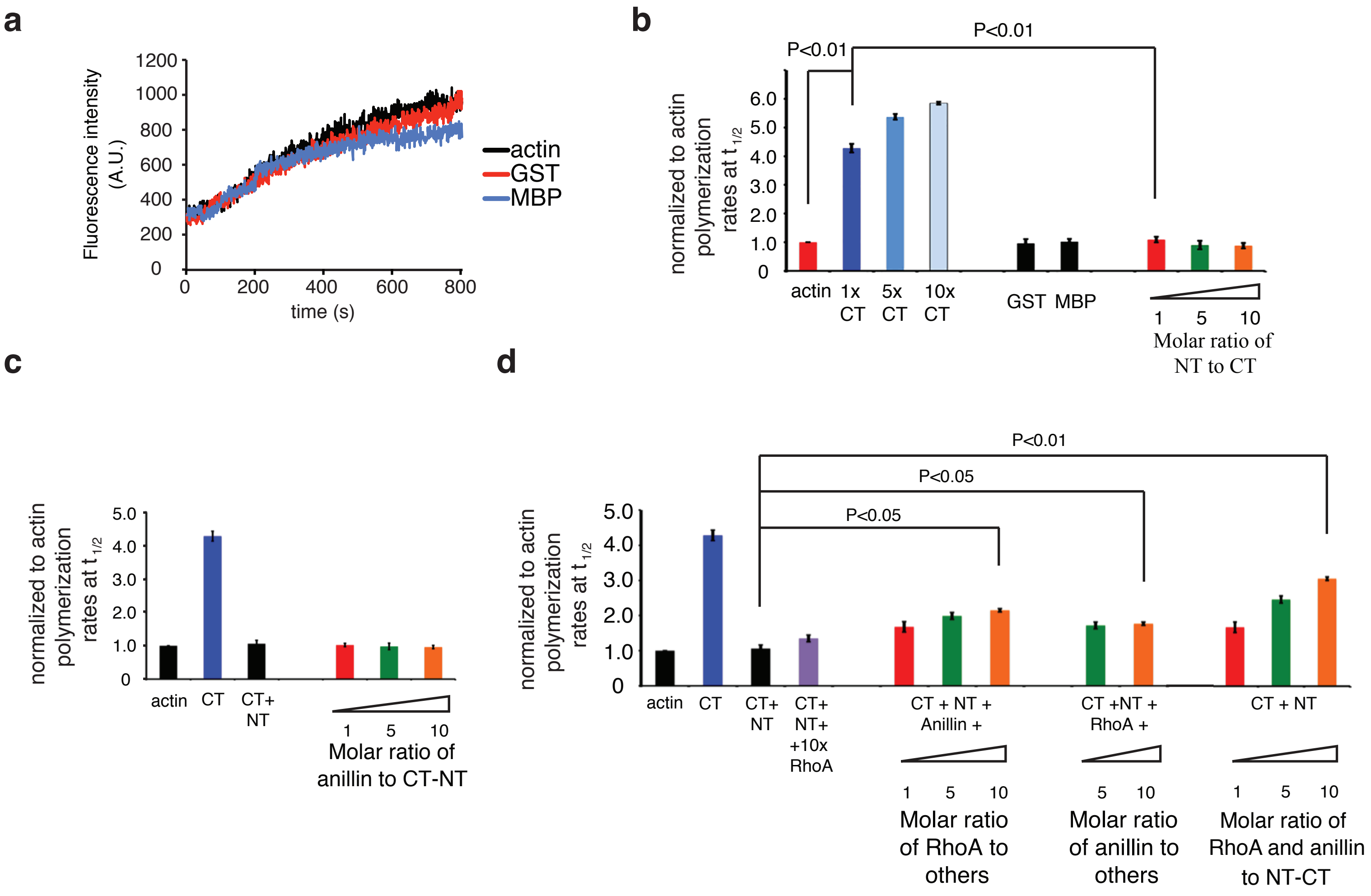


**Supplementary Figure 2.** Comparative pyrene actin polymerization assays.
**(a)** Pyrene-labeled actin polymerization rates alone and in the presence of purified recombinant MBP and GST. **(b)** Effects of different concentrations of MBP-DIAPH3 CT in the presence and absence of GST-DIAPH 3 NT on the rate of actin polymerization. Normalized actin polymerization rates at $t_{1/2}$ maximum relative to $t_{1/2}$ maximum of actin alone. n=3. (c) Effects of increasing concentration of 6xHis anillin 1-151 on actin polymerization in the presence of pre-incubated MBP-DIAPH3 CT and GST-DIAPH 3 NT. n=3. Error bars are ±s.e.m. **(d)** Effects on actin polymerization in the presence of a constant concentration of pre-incubated MBP-DIAPH3 CT and GST-DIAPH 3 NT but varying concentrations of GST RhoA, 6xHis anillin (1-151) or both. n=3. Error bars are ±s.e.m. Student's *t*-test was used for statistical analysis.

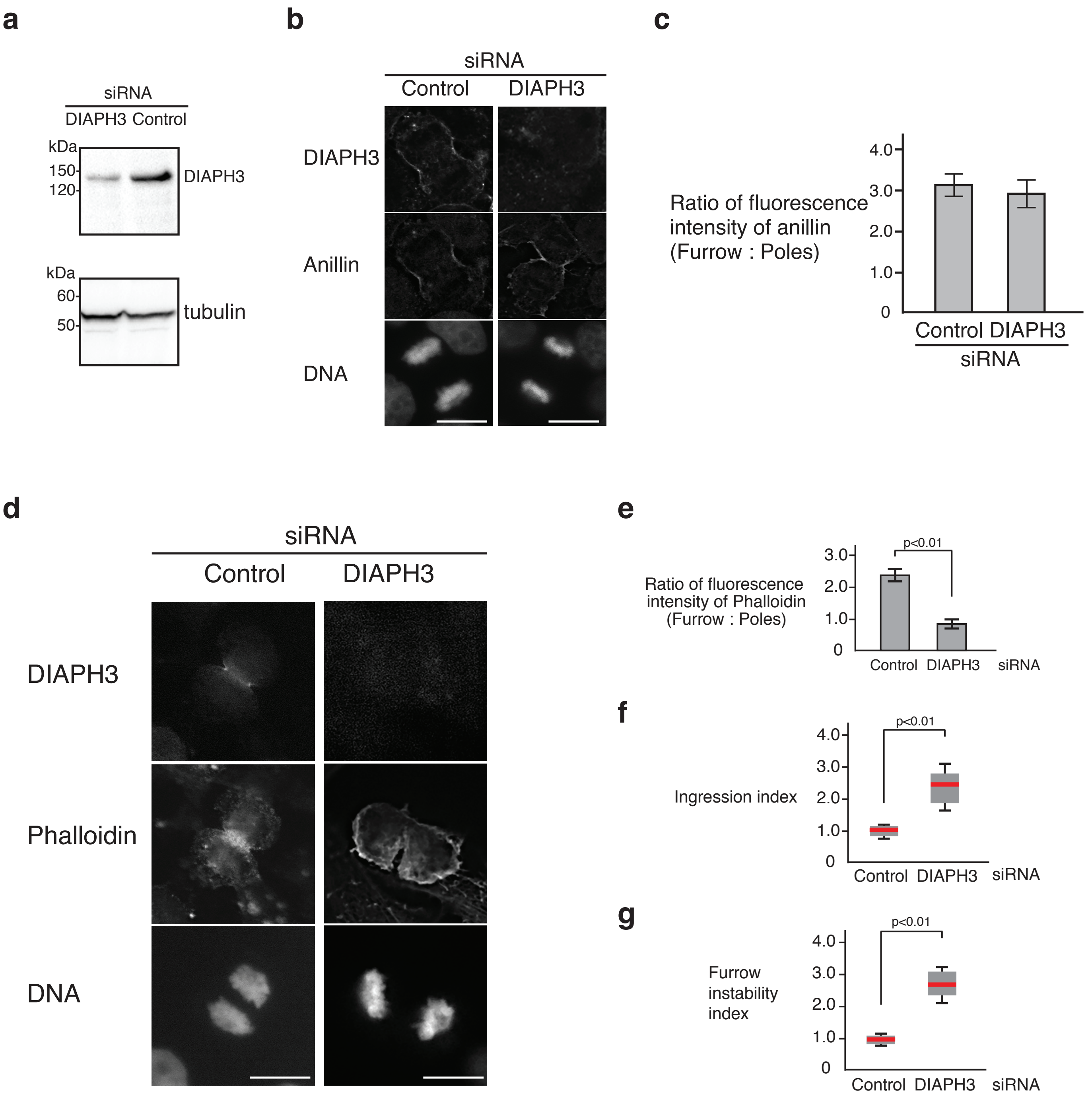


**Supplementary Figure 3.** Depletion of DIAPH3 disrupts cytokinetic actin and furrow organization. **(a)** Western blot showing the extent of DIAPH3 depletion upon siRNA treatment after 48h. **(b)** Control and DIAPH3 siRNA treated HeLa cell fixed and stained with DIAPH3, anillin antibodies and DAPI. **(c)** Quantification of the relative distribution of anillin, furrow to pole, in cells treated in b. At least 100 cells were counted in three different experiments. Error bars are ±s.e.m. **(d)** Cells treated with control and DIAPH siRNA stained with phalloidin and DAPI to determine the relative distribution of actin filaments in cytokinetic cells. **(e)** Quantification of actin distribution in cells described in e. At least 100 cells were counted in three different experiments. Error bars are ±s.e.m. **(f)** Furrow ingression index of cells treated with control and DIAPH3 siRNA. At least 100 cells were counted in three different experiments. Error bars are ±s.e.m. **(g)** Furrow stability index of cells treated with control and DIAPH3 siRNA. Error bars are ±s.e.m. Student's *t*-test was used for statistical analysis. All scale bars are 10μm.

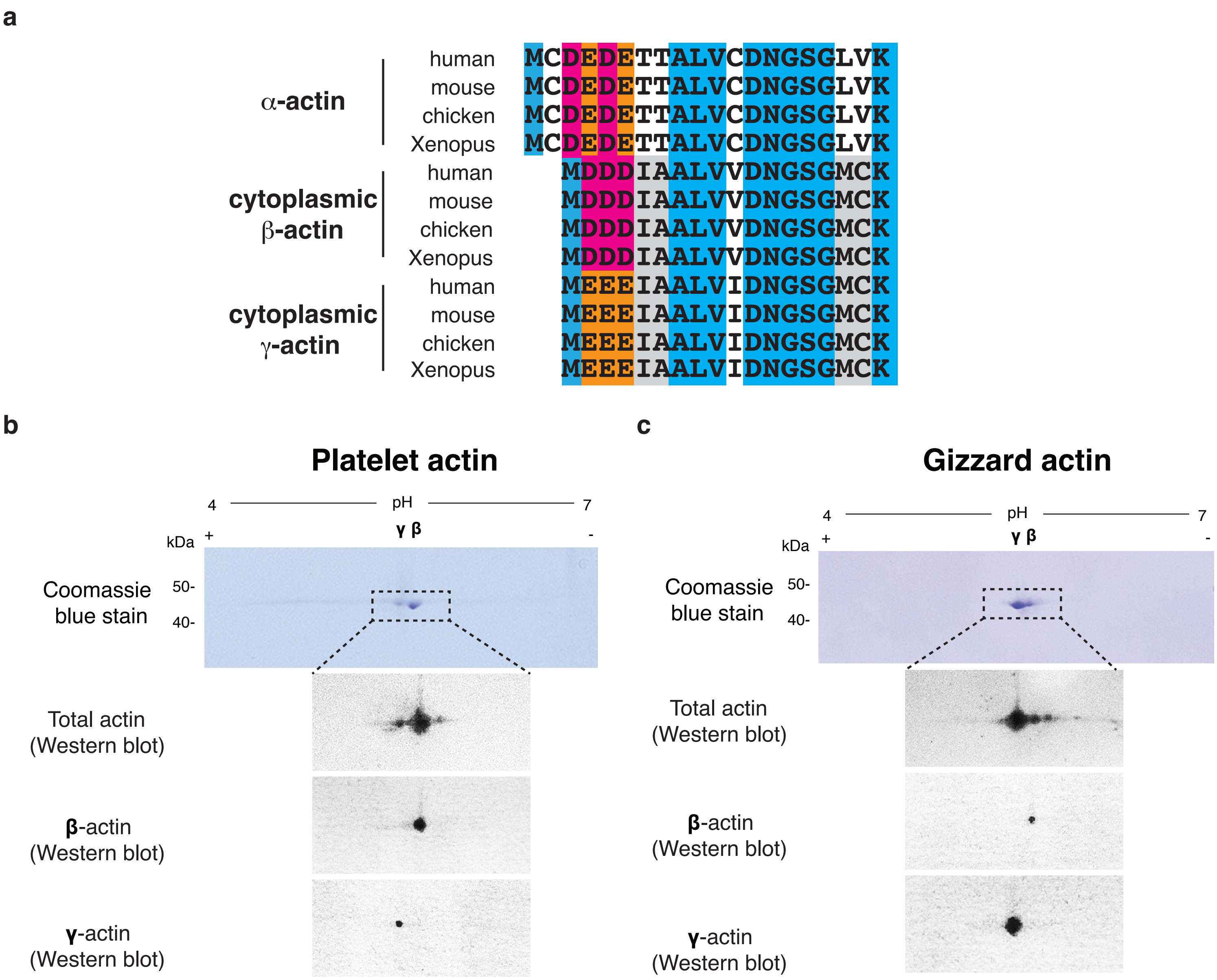


**Supplementary Figure 4.** Comparison of actin isoforms. **(a)** Cross species amino acid sequence alignment of the N-terminal 20 amino acids of different actin isoforms. Blue indicates residues conserved across isoforms and species. Pink and orange boxes highlight the divergent acidic amino acids at the N-terminus of the different actin isoforms. Gray boxes indicate residues conserved between β and γ, but not with α. **(b)** 2-D (isoelectric focusing, pH range 4-7, followed by SDS-PAGE) analysis of actin from human platelets. Top panel Coomassie stained gel, lower panels western blots probed with antibodies recognizing all actin isoforms, β-actin isoform specific and γ-actin isoform specific.
**(c)** 2-D (isoelectric focusing, pH range 4-7, followed by SDS-PAGE) analysis of actin from chicken gizzards. Top panel Coomassie stained gel, lower panels western blots probed with antibodies recognizing all actin isoforms, β-actin isoform specific and γ-actin isoform specific.

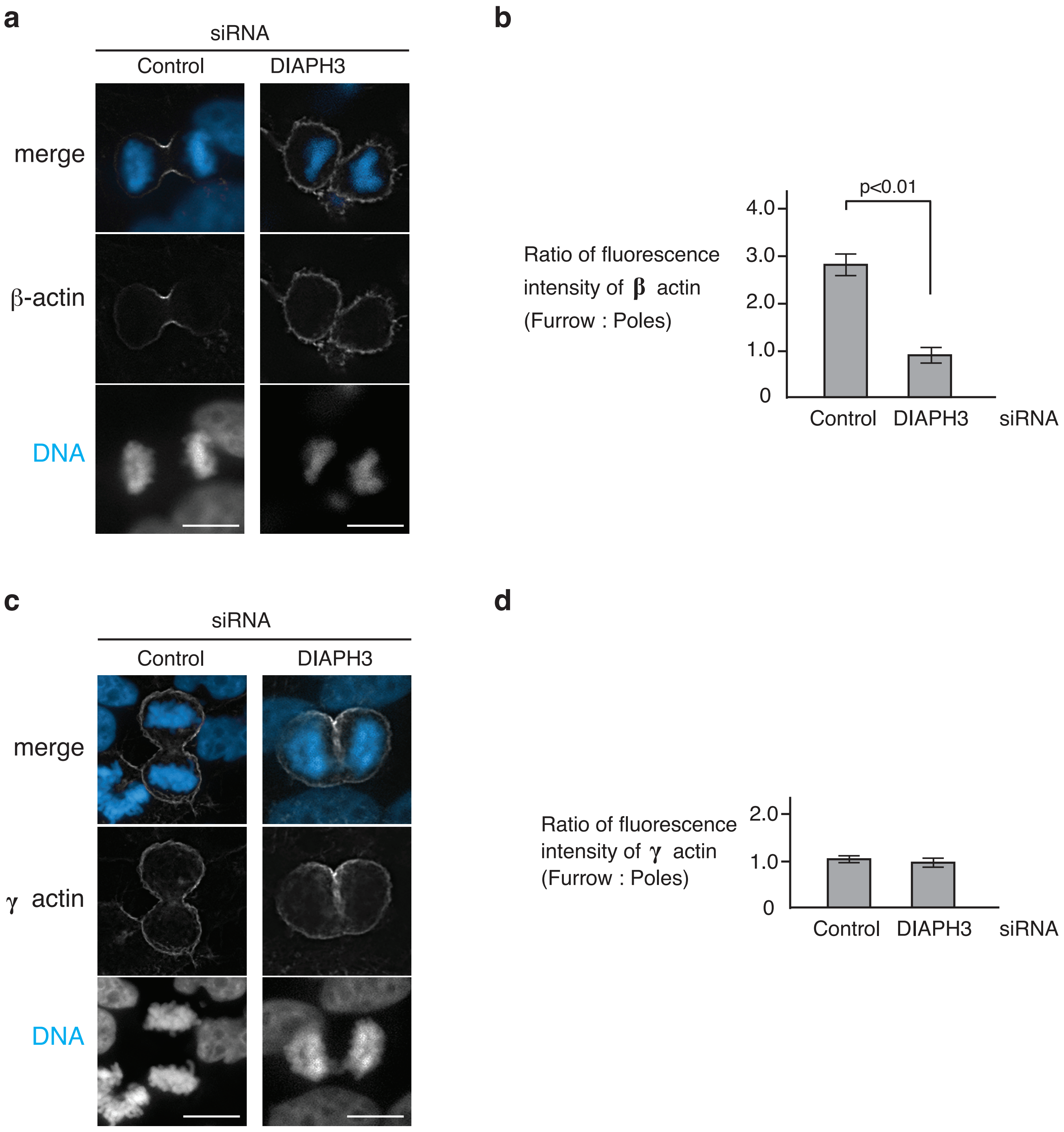


**Supplementary Figure 5.** Depletion of DIAPH3 dirupts cytokinetic β-actin organization.
**(a)** HeLa cells treated with control and DIAPH3 siRNA, fixed and stained with DIAPH3 and β-actin antibodies. **(b)** Quantification of the distribution β-actin in the cell described in a. At least 100 cells were counted in three different experiments. Error bars are ±s.e.m. Student's *t*-test was used.
**(c)** HeLa cells treated with control and DIAPH3 siRNA, fixed and stained with DIAPH3 and γ-actin antibodies. **(d)** Quantification of the distribution γ-actin in the cell described in c. At least 100 cells were counted in three different experiments. Error bars are ±s.e.m. All scale bars are 10μm.

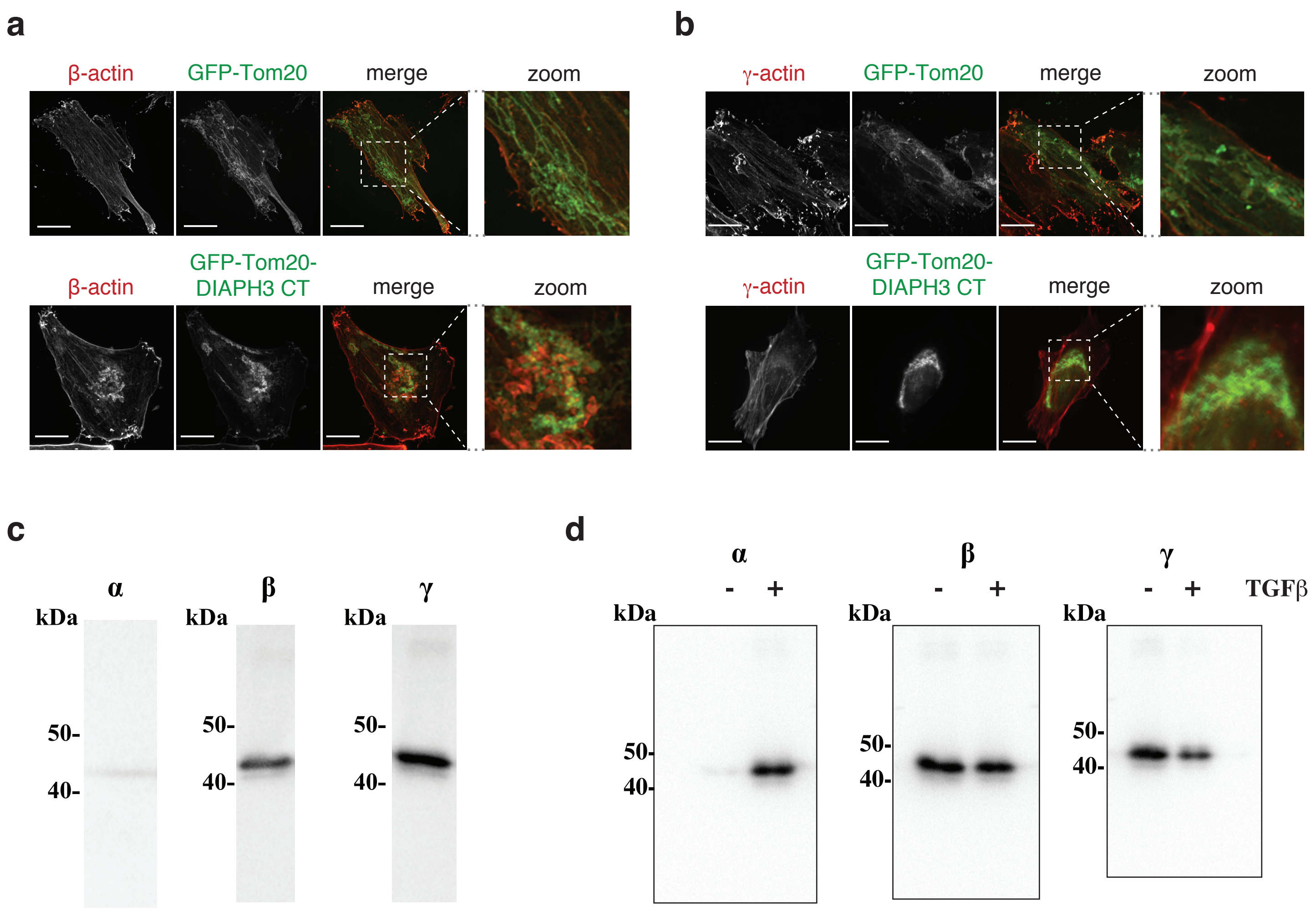


**Supplementary Figure 6.** Ectopic targeting of DIAPH3 to the surface of mitochondria stimulates the localized production of β-actin but not γ-actin. The C-terminal region of DIAPH3 that contains the FH1 domain and the FH2 actin nucleation domain was fused to GFP and the mitochondrial-targeting element of TOM20 then transfected into HeLa cells. Cells were fixed 24 hours later and stained with antibodies that specifically recognize **(a)** β- and **(b)** γ-actin. Scale bars are 10µm. **(c)** Immunoblot of HeLa cell lysates probed with α-, β- and γ-actin specific antibodies. **(d)** Immunoblot of MRC-5 cell lysates, generated from cells grown in the absence or presence of 2ng/ml TGFβ for 96 hours to induce the expression of α-actin, probed with α-, β- and γ-actin specific antibodies.

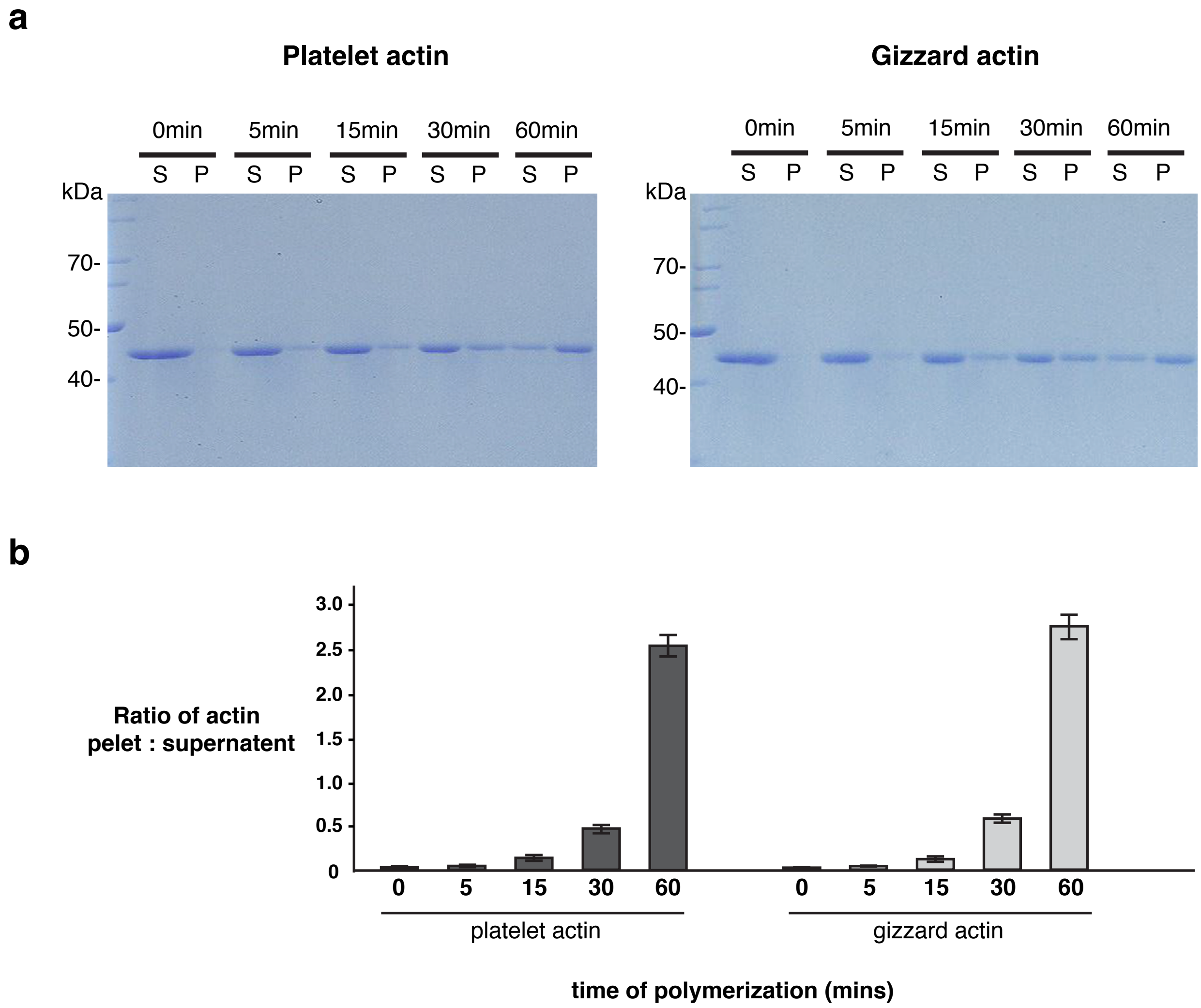


**Supplementary Figure 7.** Comparative polymerization activity of actin isolated from human platelets and chicken gizzards. Actin from the different sources was incubated in polymerization buffer and at different time points centrifuged at 65,000rpm for 20min in a TLA 120.2 rotor applied in Beckman TL-100 Ultracentrifuge (Beckman Coulter, Inc.). **(a)** The amount of actin in the supernatant (S), unpolymerized actin was compared to the amount of actin in the pellet (P), polymerized actin by SDS-PAGE and coomassie blue staining. **(b)** The comparative amount of actin in the pellet and supernatant fractions was determined by densitometry measurements of the Coomassie blue stained SDS PAGE gels in a. n=3. Error bars are ±s.e.m.

## Figure 1 blots

### Figure 1b

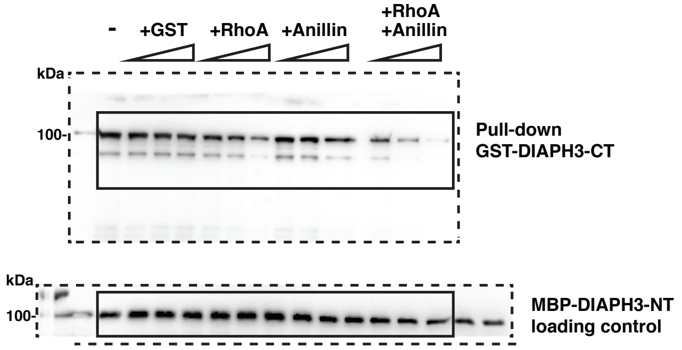


### Figure 1c

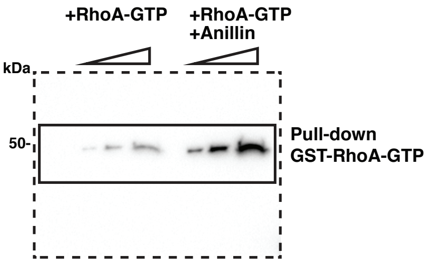


### Figure 1d

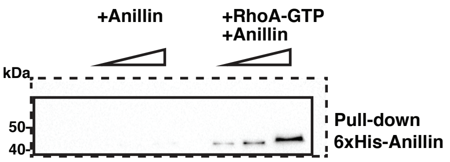


## Figure 2 blots

### Figure 3a

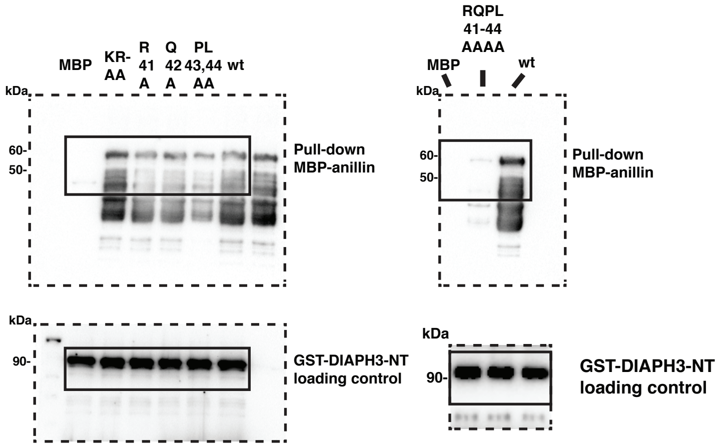


### Figure 3c

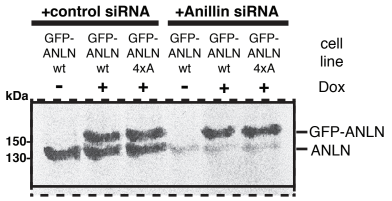


### Figure 3d

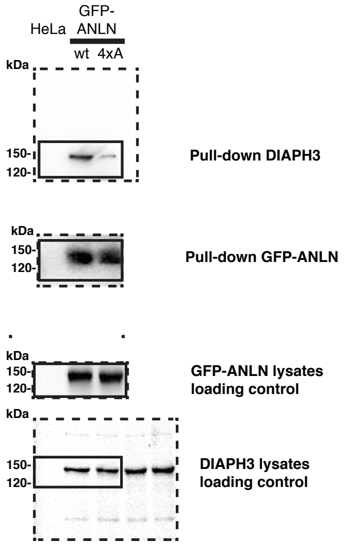


## Figure 5 blots

### Figure 5a

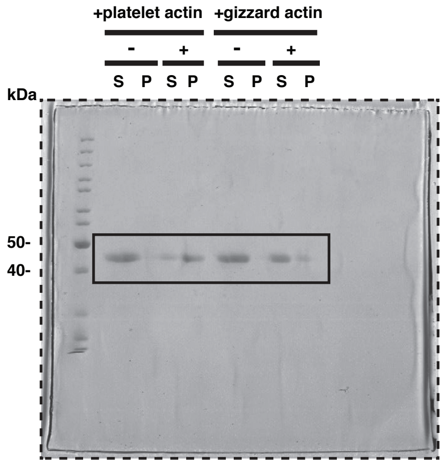


### Figure 5b

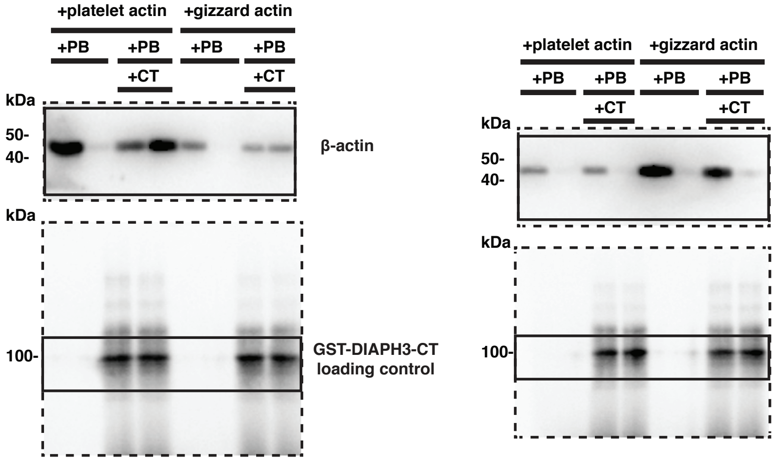


**Supplementary Figure 8.** Uncropped images of all blots in Figures 1, 3 and 5

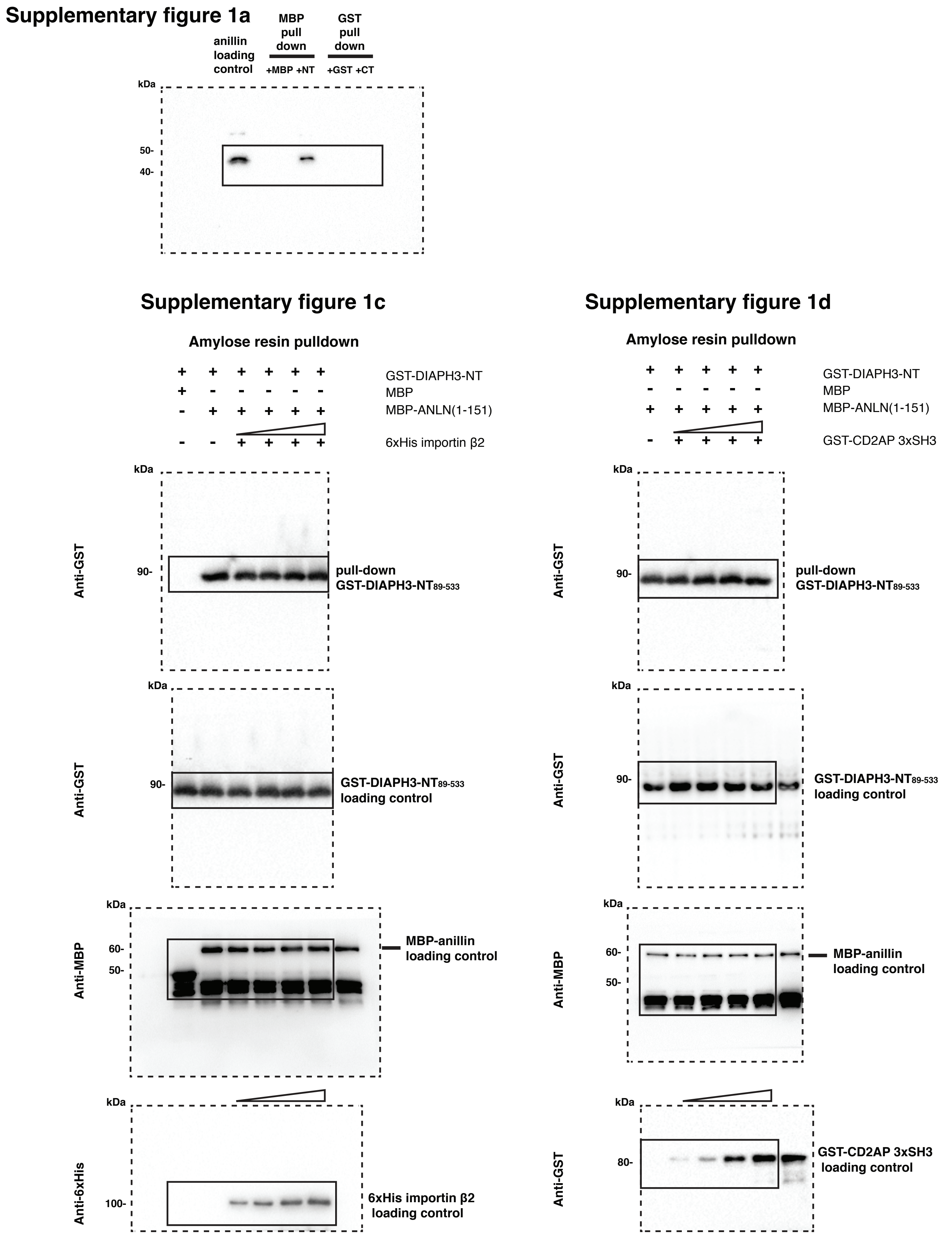


**Supplementary Figure 9.** Uncropped images of blots in Supplementary Figure 1

## Supplementary Figure 3 blots

### Supplementary figure 3a

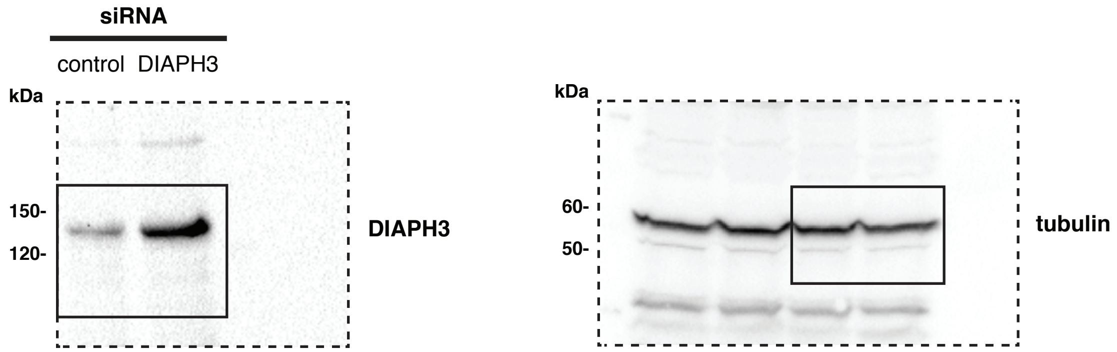


## Supplementary Figure 4 blots

### Supplementary figure 4b

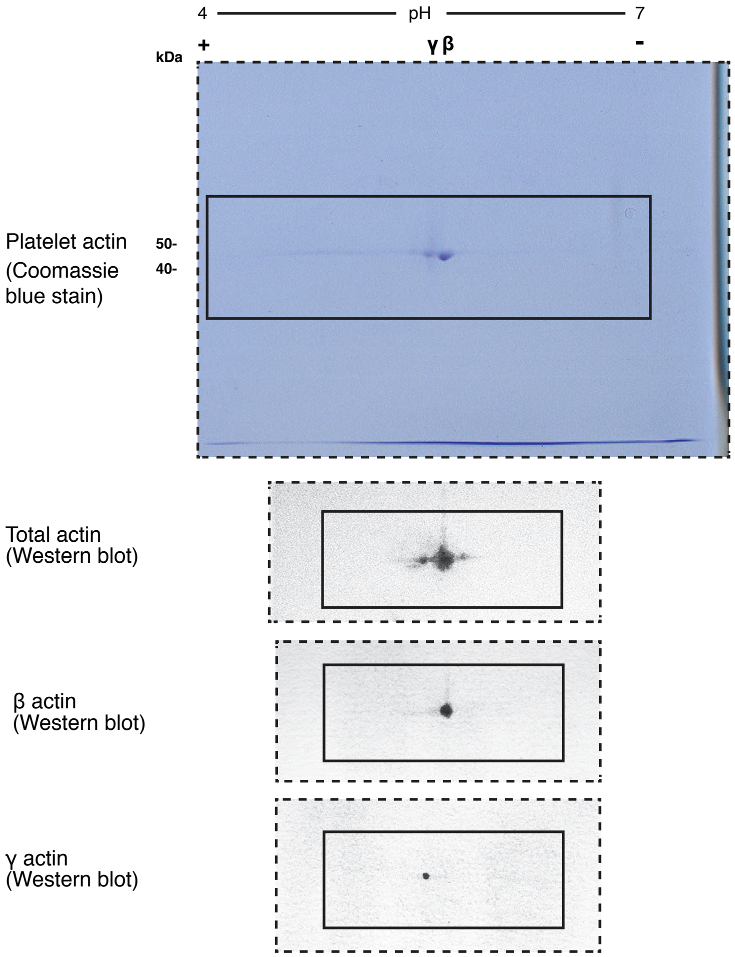


### Supplementary figure 4c

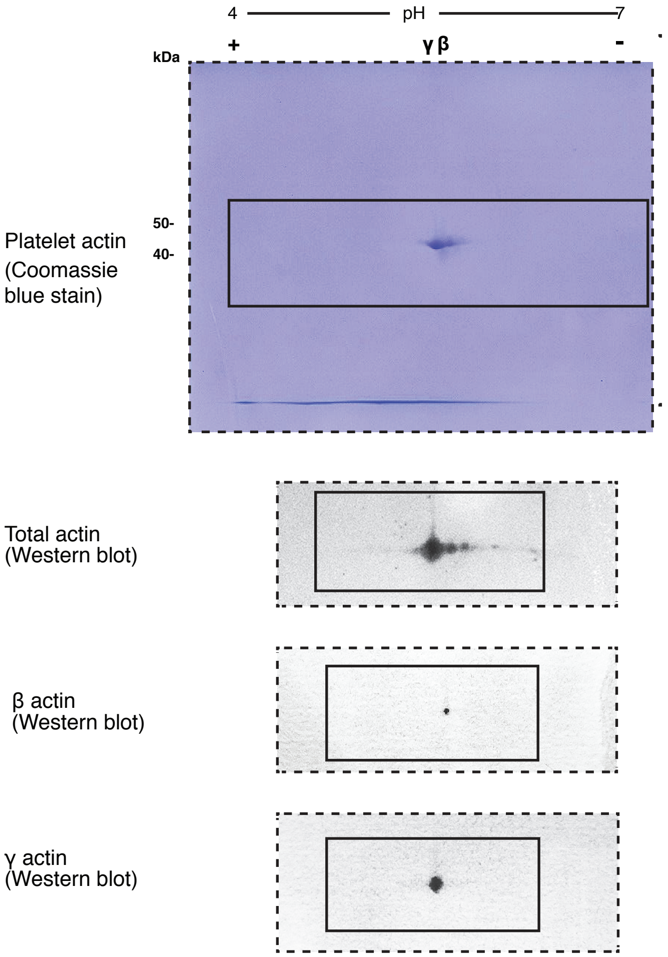


**Supplementary Figure 10** Uncropped blots and gels from Supplementary Figures 3 and 4

## Supplementary Figure 6c

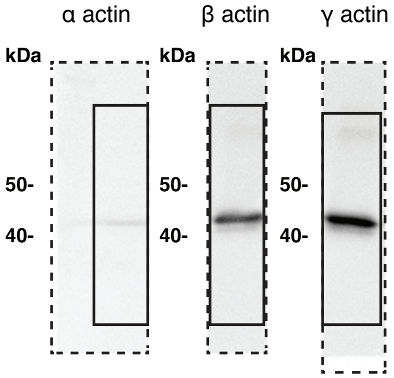


## Supplementary Figure 7 gels

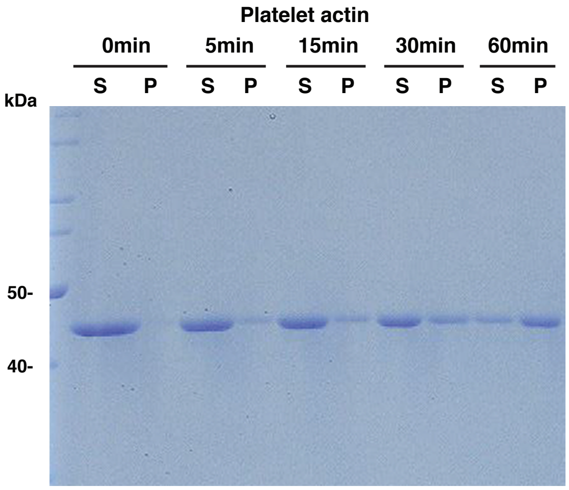


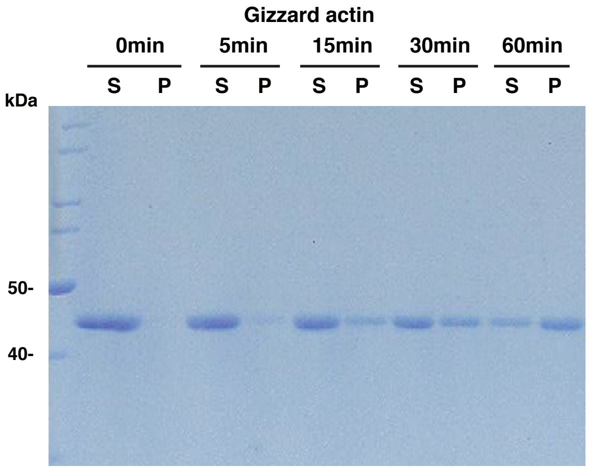


**Supplementary Figure 11.** Uncropped blots and gels from Supplementary Figs 6 and 7

**Supplementary Table 1: Applied DNA oligonucleotides**

| Name | Sequence (5' to 3') |
|---|---|
| 5'_anillin | ATGGATCCGTTTACGGAGA |
| 3'_anillin | TTAAGGCTTCCCAACAGGCTT |
| 5'_anillln_Lic | GCTGGCGCTGGTGCGGGTGCCGGAATGGATCCGTTTACGGA |
| 3'_anillin_Lic | TCCGTGGCGGCCTCGTCGTCGGGATTAAGGCTTCCCAACAGGC |
| fwd_anilln_22 | AAAATGGCTGAGAGGCCCA |
| fwd_ anillin _57 | GAGAAATCTTGTACAAAACC |
| rev_ anillin _57 | TTCACCACCAGAGAGGGG |
| rev_ anillin _91 | TGTCGACTCAACTGGTTGTTT |
| rev_ anillin _151 | CTCTGCAAGTTTTTGCATAC |
| rev_ anillin _300 | AGTAGATTTCACTGGAGAAG |
| 5'_mDia2 | ATGGAACGGCACCAGCCG |
| 3'_mDia2 | TTATAAAGCTCGTAATCTTGCC |
| fwd_mDia2_89 | CCACTTCCCAACCTGAAG |
| fwd_mDia2_561 | CCTTTGCCTCCCTCTAAAGA |
| rev_ mDia2_533 | TTTTTTCTGCAATTCAGCCTGAGT |
| fwd_anillin_ KR (38-39)-AA | CCAAGGTCTATGACTCATGCTGCAGCAGCTAGACAGCCA |
| rev_anillin_ KR (38-39)-AA | TTCTGAAAGTGGCTGTCTAGCTGCTGCAGCATGAGTCAT |
| fwd_anillin_R41A | ATGACTCATGCTAAGCGAGCTGCACAGCCACTTTCAGAA |
| rev_anillin_R41A | TGCTTCTGAAAGTGGCTGTGCAGCTCGCTTAGCATGAGT |
| fwd_anillin_Q42A | ACTCATGCTAAGCGAGCTAGAGCACCACTTTCAGAAGCAA |
| rev_anillin_Q42A | GTTACTTGCTTCTGAAAGTGGTGCTCTAGCTCGCTTAGCATG |
| fwd_anillin_ PL (43-44)-AA | CATGCTAAGCGAGCTAGACAGGCAGCATCAGAAGCAAGTAAC |
| rev_anillin_ PL (43-44)-AA | CTGCTGGTTACTTGCTTCTGATGCTGCCTGTCTAGCTCGCT |
| fwd_anillin_ RQPL (41-44)-AAAA | TCTATGACTCATGCTAAGCGAGCTGCAGCAGCAGCATCAGAAG CAAGTAAC |
| rev_anillin_ RQPL (41-44)-AAAA | GGGCTGCTGGTTACTTGCTTCTGATGCTGCTGCTGCAGCTCGCT TAGCATGAGT |
| fwd_Tom20-GFP-CT | ATGGACGAGCTGTACAAGAAACTAGAAGAGTTTGAAGAG |
| rev_Tom20-GFP-CT | TCTTGAGTCGCGGCCTTATAAAGCTCGTAATCTTGC |

**fwd**: forward primer **rev**: reverse primer **Lic**: Ligation independent cloning

**CT**: C-terminus of mDia2

## Supplementary Table 2: Applied siRNA oligonucleotides

| Name | Sequence (5' to 3') |
|---|---|
| 3'UTR anillin | AGCUUACAGACUUAGCAU |
| NC | CGUUAAUCGCGUAUAAUACGCGUT |
| 3' UTR DIAPH3 | CCUUAAUUGGGAUCUAUAUACUACC |

**UTR**: Untranslated region **NC**: Negative control
