# Supplementary material for: Cytokinesis requires localized β-actin filament production by an actin isoform specific nucleator
Source: Nat Commun. 2017 Nov 16;8:1530. doi: 10.1038/s41467-017-01231-x (PMC5691081; doi:10.1038/s41467-017-01231-x)
Supplement: Supplementary file 2 — Description of Additional Supplementary Files [file 41467_2017_1231_MOESM2_ESM.pdf]

Description of Additional Supplementary Files

File Name: Supplementary Movie 1

Description: Early cytokinesis of a HeLa cell stably expressing GFP-anillin.

File Name: Supplementary Movie 2

Description: Early cytokinesis of a HeLa cell stably expressing GFP-anillin that has been treated with anillin siRNA to deplete endogenous anillin.

File Name: Supplementary Movie 3

Description: Early cytokinesis of a HeLa cell stably expressing a GFP-anillin mutant, anillin 41 RQPLAAAA 44, that prevents the binding of anillin to DIAPH3. This cell has also been treated with anillin siRNA to deplete endogenous anillin.
